# Decentralized Analysis of Brain Imaging Data: Voxel-Based Morphometry and Dynamic Functional Network Connectivity

**DOI:** 10.3389/fninf.2018.00055

**Published:** 2018-08-27

**Authors:** Harshvardhan Gazula, Bradley T. Baker, Eswar Damaraju, Sergey M. Plis, Sandeep R. Panta, Rogers F. Silva, Vince D. Calhoun

**Affiliations:** ^1^The Mind Research Network, Albuquerque, NM, United States; ^2^Department of Computer Science, The University of New Mexico, Albuquerque, NM, United States; ^3^Department of Electrical and Computer Engineering, The University of New Mexico, Albuquerque, NM, United States

**Keywords:** decentralized algorithms, COINSTAC, VBM, dFNC, multi-shot

## Abstract

In the field of neuroimaging, there is a growing interest in developing collaborative frameworks that enable researchers to address challenging questions about the human brain by leveraging data across multiple sites all over the world. Additionally, efforts are also being directed at developing algorithms that enable collaborative analysis and feature learning from multiple sites without requiring the often large data to be centrally located. In this paper, we propose two new decentralized algorithms: (1) A decentralized regression algorithm for performing a voxel-based morphometry analysis on structural magnetic resonance imaging (MRI) data and, (2) A decentralized dynamic functional network connectivity algorithm which includes decentralized group ICA and sliding-window analysis of functional MRI data. We compare results against those obtained from their pooled (or centralized) counterparts on the same data i.e., as if they are at one site. Results produced by the decentralized algorithms are similar to the pooled-case and showcase the potential of performing multi-voxel and multivariate analyses of data located at multiple sites. Such approaches enable many more collaborative and comparative analysis in the context of large-scale neuroimaging studies.

## 1. Introduction

In the current times, innovation and discovery are often underpinned by the size of data at one's disposal and this has led to a paradigm shift in scientific research increasing the emphasis on collaborative data-sharing (Cragin et al., 2010; Tenopir et al., 2011). This growing significance of data-sharing is more evident in the field of neuroscience where, in the past few years, there has been a proliferation of efforts (Poldrack et al., 2013) toward enabling researchers to leverage data across multiple sites. In part, this is due to the fact that collecting neuroimaging data is expensive as well as time consuming (Landis et al., 2016) and aggregating or sharing data across various sites provides researchers with an opportunity to uncover important findings that are beyond the scope of the original study (Poldrack et al., 2013). In addition to making predictions more certain by increasing the sample size (Button et al., 2013), sharing data ensures reliability and validity of the results, and safeguards against data fabrication and falsification (Tenopir et al., 2011; Ming et al., 2017).

As mentioned previously, data-specific collaborative efforts include either aggregating the data via a centralized data sharing repository or sharing data via agreement based collaborations, or data usage agreement (DUA) in other words (Thompson et al., 2014, 2017). However, each methodology has its own set of barriers. For example, policy or proprietary restrictions or data re-identification concerns (Sweeney, 2002; Shringarpure and Bustamante, 2015) might hinder data sharing whereas DUAs might take months to complete and even if one comes through, there is no guarantee of the utility of the data until the planned analysis is performed (Baker et al., 2015; Ming et al., 2017). Other significant challenges include the storage and computational resources needed which could prove costly as the volume of the data shared goes up.

Frameworks such as ENIGMA (Thompson et al., 2014, 2017) to some extent bypass the need for DUAs by performing a centrally coordinated analysis at each local site. This enables potentially large data at each local site to stay put allowing a greater level of control as well as privacy. Another framework called ViPAR (Carter et al., 2015) tries to go one step further by, relying on open-source technologies, completely isolating the data at the local site but only pooling them via transfer to perform automated statistical analyses. This repeated pooling of data becomes cumbersome as the number of sites or the size of the data at each site goes up and ENIGMA (Thompson et al., 2014, 2017; Hibar et al., 2015; van Erp et al., 2016) addresses this issue by pooling local statistical results for further analysis, also known as, meta-analysis (Adams et al., 2016). However, the heterogeneity among the local analyses caused by adopting various data collection mechanisms or preprocessing methods can lead to inaccurate meta-analysis findings.

Plis et al. (2016), proposed a web-based framework titled Collaborative Informatics and Neuroimaging Suite Toolkit for Anonymous Computation (COINSTAC) to address the aforementioned issues. COINSTAC provides a platform to analyze data stored locally across multiple organizations without the need for pooling the data at any point during the analysis. It is intended to be an ultimate one-stop shop by which researchers can build any statistical or machine learning model collaboratively in a decentralized fashion. This framework implements a message passing infrastructure that will allow large scale analysis of decentralized data with results on par with those that would have been obtained if the data were in one place. Since, there is no pooling of data it also preserves the privacy of individual datasets.

Some of the decentralized computations discussed in the literature so far include decentralized regression (Plis et al., 2016), joint independent component analysis (Baker et al., 2015), decentralized independent vector analysis (Wojtalewicz et al., 2017), decentralized neural networks (Lewis et al., 2017), decentralized stochastic neighbor embedding (Saha et al., 2017) and many more. To our knowledge, most of these algorithms have been tested on synthetic data. In this work we present two new decentralized algorithms that are widely used in a centralized manner in the imaging community and demonstrate their utility on real world brain imaging data.

Regression, is widely used in neuroimaging studies as it enables one to regress certain covariates, for example- age, diagnosis, gender or treatment response, to study their effects on the structure and function of various brain regions. Some examples of regression related studies in this field include (Fennema-Notestine et al., 2007) where regression was used as a validity test in examining the aggregation of structural imaging across different datasets. In addition, the very successful ENIGMA studies are mostly using regression analyses for a small number of variables. Roshchupkin et al. (2016) presented a framework titled HASE (high-dimensional association analyses) that is capable of analyzing high-dimensional data at full resolution, yielding exact association statistics. While singleshot and multishot regression have been presented previously (Plis et al., 2016), their treatment was cursory in nature without any actual consideration of the appropriate gradient descent scheme or the validity of the methods on real datasets both of which have been presented in this work.

In this paper, in addition to improving the single-shot and multi-shot regression we also present a new variant of decentralized regression- “decentralized regression with normal equation” and extend this work to operate on voxels in an MRI image, in order to implement a voxel-based morphometry (VBM) study in a decentralized framework (Ashburner and Friston, 2000). We implement and evaluate the proposed decentralized VBM approach on the publicly available MIND Clinical Imaging Consortium (MCIC) dataset (available via the COINS data exchange at https://coins.mrn.org and contrast the results obtained with those from pooled/centralized regression to validate the proof-of-concept.

Another widely utilized method in neuroimaging analysis is dynamic functional network connectivity (dFNC) (Sakoglu et al., 2010; Allen et al., 2014). dFNC is an analysis pipeline for functional magnetic resonance imaging (fMRI) data, which allows for the identification and analysis of networks of co-activating brain states. In contrast to static approaches (Smith et al., 2009), which take the mean connectivity over time-points, dFNC uses clustering of time varying connectivity estimates computed from sliding-windows taken over subject time-courses, thus becoming desirable in experiments where network connectivity is highly dynamic in the time dimension, for example in experiments which utilize resting-state fMRI (Deco et al., 2013; Damaraju et al., 2014).

Importantly, dFNC is focused on time-courses of networks extracted from a group independent component analysis (ICA), which is a widely used approach for estimating functional brain networks (Calhoun and Adali, 2012) and as such to implement dFNC we needed to also implement a decentralized group ICA approach.

For collaborative neuroimaging applications, a decentralized version of dFNC is desirable for many of the same reasons as regression, and currently, no such decentralized version exists. Unlike regression, however, the dFNC pipeline consists of multiple, distinct stages, all of which require decentralization. In this paper, we present an initial version of decentralized dFNC by providing decentralized approaches to both the group spatial independent component analysis (ICA) and K-Means clustering steps in the pipeline, which, along with additional preprocessing steps including sliding window correlation, can be implemented together to perform decentralized dFNC. Our resulting methods, dgICA, and ddFNC via dK-Means, provide dynamic connectivity results consistent with established pooled approaches in the literature, thus representing an important step toward more exhaustive analysis of the decentralized approaches to the dFNC pipeline. Our contributions in this paper can thus be summarized as follows.

1. Development of decentralized regression with normal equation, improvement of single-shot and multi-shot regression and their validation on structural MRI data
2. Presentation of a decentralized dynamic functional network connectivity analysis pipeline and its evaluation on functional MRI data

## 2. Methods

### 2.1. Decentralized VBM (i.e., voxelwise decentralized regression)

Statistical analysis plays a key role in the field of neuroimaging studies. Researchers would often want to characterize the effect of various factors such as age, gender, disease condition, etc., on the composition of brain tissue at various regions of the brain. Voxel-based morphometry (VBM) (Ashburner and Friston, 2000) is one such approach that facilitates a comprehensive comparison, via generalized linear modeling, of voxel-wise gray matter concentration between different groups, for example. To enable such statistical assessment on data present at various sites, it is important to develop decentralized tools. In this section, we first provide a brief overview of decentralized regression algorithms (the building blocks of decentralized VBM which is essentially voxel-wise regression) along with some notation.

The goal of decentralized regression is to fit a linear equation (given by Equation 1) relating the covariates at *S* different sites to the corresponding responses. Assume each site *j* has data set $D_{j}=\left\{\left({\text{x}}_{i},y_{i}\right):i\in \left\{1,2,\ldots ,s_{j}\right\}\right\}$ where ${\text{x}}_{i,j}\in R^{d}$ is a *d*-dimensional vector of real-values features, and *y*_*j*_∈ is a response. We consider fitting the model in Equation 2 where **w** is given as [**w**; *b*] and **x** as [**x**; 1]

(1) $$\begin{array}{r} y\approx {\text{w}}^{⊤}\text{x}+b \end{array}$$

(2) $$\begin{array}{r} \text{y}\approx {\text{w}}^{⊤}\text{x} \end{array}$$

The vector of regression parameters/weights **w** is found by minimizing the sum of the squared error given in Equation (3)

(3) $$\begin{array}{r} F\left(\text{w}\right)=\overset{S}{\underset{j=1}{\sum }}\overset{s_{j}}{\underset{i=1}{\sum }}{\left(y_{i}-{\text{w}}^{⊤}{\text{x}}_{i,j}\right)}^{2} \end{array}$$

The regression objective function is a linearly separable function, that can be written as sum of a local objective function calculated at each local site as follows:

(4) $$\begin{array}{r} F\left(\text{w}\right)=\overset{S}{\underset{j=1}{\sum }}F_{j}\left(\text{w}\right) \end{array}$$

where

(5) $$\begin{array}{r} F_{j}\left(\text{w}\right)=\overset{s_{j}}{\underset{i=1}{\sum }}\left(y_{i}-{\text{w}}^{⊤}{\text{x}}_{i,j}\right) \end{array}$$

A central aggregator (*AGG*) is assumed whose role is to compute the global minimizer $\hat{\text{w}}$ of *F*(**w**).

#### 2.1.1. Single-shot regression

In one approach to solve the decentralized regression problem, termed the *single-shot* regression (Plis et al., 2016), each site *j* finds the minimizer ${\hat{\text{w}}}_{j}$ of the local objective function *F*_*j*_(**w**). This is the same as solving the regression problem at each local site. Once the regression model at each site is fit, the weights are sent to the central aggregator (*AGG*) where they are aggregated (weighted average) to find the global minimizer or can be used separately to perform a meta-analysis similar to those performed in ENIGMA (using a manual spreadsheet-based approach however) (Turner et al., 2013; van Erp et al., 2016). The pseudocode to perform single-shot decentralized regression (Plis et al., 2016), with a slight modification, is presented here again for completeness.

**Algorithm 1:**
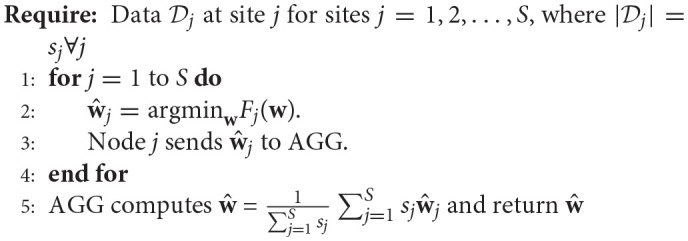
Single-shot Regression

#### 2.1.2. Decentralized regression with normal equation

One limitation of single-shot regression is that the “site” level covariates cannot be included at each local site as this leads to collinearity issues. This issue can be offset by utilizing a decentralized version of the analytical solution to the linear regression problem. For a standard regression problem of the form given in Equation (2), the analytical solution is given as

(6) $$\begin{array}{r} \hat{\text{w}}={\left({\text{x}}^{⊤}\text{x}\right)}^{-1}{\text{x}}^{⊤}\text{y} \end{array}$$

Assuming that the augmented data matrix **x** is made up of data from different local sites, i.e.,

(7) $$\begin{array}{r} \text{x}=\left[\begin{array}{c} {\text{x}}_{1}\\ \vdots \\ {\text{x}}_{S} \end{array}\right] \end{array}$$

it's easy to see that $\hat{\text{w}}$ can be written as

(8) $$\begin{array}{l} \hat{w}={\left(\left[\begin{array}{ccc} x_{1}^{⊤} & \cdots & x_{S}^{⊤} \end{array}\right]\left[\begin{array}{c} x_{1}\\ \vdots \\ x_{S} \end{array}\right]\right)}^{-1}\times \\ \;\left[\begin{array}{ccc} x_{1}^{⊤} & \cdots & x_{S}^{⊤} \end{array}\right]\left[\begin{array}{c} y_{1}\\ \vdots \\ y_{s} \end{array}\right] \end{array}$$

(9) $$\hat{w}={\left(\sum_{j=1}^{S}x_{j}^{T}x_{j}\right)}^{-1}\times \left(\sum_{j=1}^{S}x_{j}^{T}y_{j}\right)$$

The above variant of the analytical solution to a regression model shows that even if the data resides in different locations, fitting a global model in the presence of site covariates delivers results that are exactly similar to the pooled case.

**Algorithm 2:**
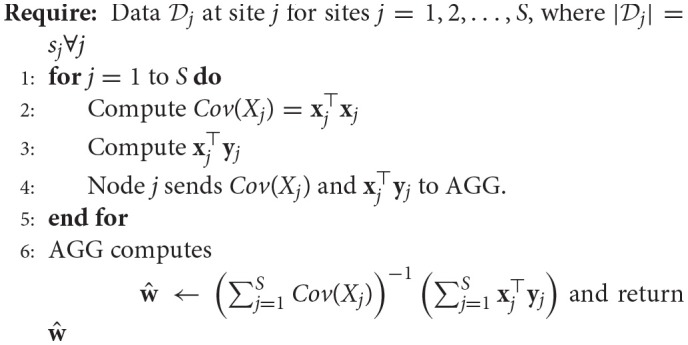
Decentralized Regression with Normal Equation

#### 2.1.3. Multi-shot regression

Decentralized regression with a normal equation is a nice mathematical formulation which produces results that are exactly the same as those from the pooled regression. However, one of the biggest drawback of the analytical form of regression is it becomes computationally expensive to evaluate the inverse of **x**^⊤^**x** as the number of features in the dataset (D) increases. While in a neuroimaging setting there might not be as many covariates to make it computationally expensive, it is indeed a challenge while working with datasets where the cardinality of the feature set is usually large (especially in machine learning). One can overcome this drawback by implementing an optimization method in a way that entails the local sites and *AGG* having to communicate iteratively. This is a type of distributed gradient descent and such a regression is termed “*multi-shot*” regression (Plis et al., 2016).

For a regression model of the form given in Equation 5, the gradient update equation (given a learning rate η) is given as

(10) $$\begin{array}{r} {\hat{\text{w}}}_{t+1}={\hat{\text{w}}}_{t}-\eta \cdot ▿F_{j}\left(\hat{\text{w}}\right) \end{array}$$

**Algorithm 3:**
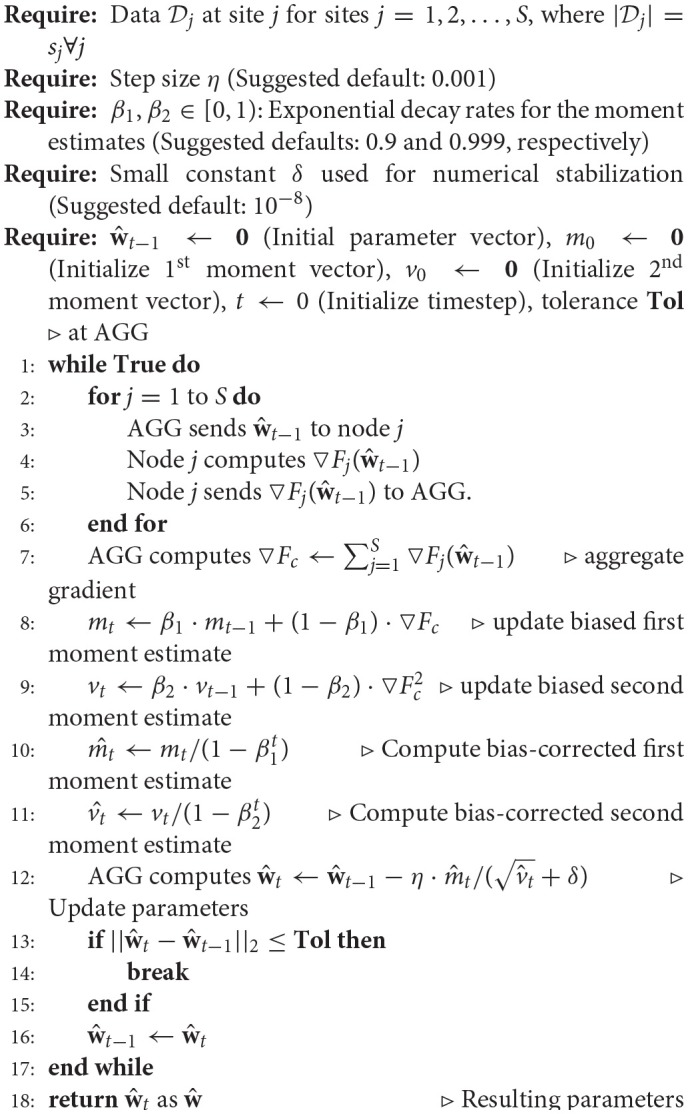
Multi-shot Regression

where

(11) $$\begin{array}{r} ▿F_{j}\left(\hat{\text{w}}\right)=\overset{s_{j}}{\underset{i=1}{\sum }}\left(y_{i}-{\hat{\text{w}}}^{⊤}{\text{x}}_{i,j}\right){\text{x}}_{i,j} \end{array}$$

In multi-shot regression, at every time step the *AGG* sends the value of $\hat{{\text{w}}_{t-1}}$ to each of the local sites which then compute their local gradients ▿*F*_*j*_(*w*_*t*_) and send them back to the *AGG* where it sums up all the local gradients in order to update the parameter vector $\hat{{\text{w}}_{t}}$. The need to sum up all the local gradients is explained as follows:

(12) $$\begin{array}{l} \text{From Equation }(\text{4}),\text{ F}(\hat{w})\text{=}\sum_{\text{j=1}}^{\text{S}}{\text{F}}_{j}(\hat{w})\\ \;∴▽\text{F}(\hat{w})\text{= }\sum_{\text{j=1}}^{\text{S}}▽{\text{F}}_{j}(\hat{w})\; \end{array}$$

To illustrate this using an example, suppose there are 3 sites (*S* = 3) with *s*_1_, *s*_2_ and *s*_3_ number of samples, respectively, at each site. The global objective function $F\left(\hat{\text{w}}\right)$ can be easily written as the sum of objective functions from each site (this because the objective function is linear) as follows:

(13) $$\begin{array}{l} F(\hat{w})=\sum_{j=1}^{s_{1}+s_{2}+s_{3}}{\left(y_{j}-{\hat{w}}^{⊤}x_{j}\right)}^{2}\\ \;=\sum_{j=1}^{s_{1}}{\left(y_{j}-{\hat{w}}^{⊤}x_{j}\right)}^{2}+\sum_{j=1}^{s_{2}}{\left(y_{j}-{\hat{w}}^{⊤}x_{j}\right)}^{2}\\ \;+\sum_{j=1}^{s_{3}}{\left(y_{j}-{\hat{w}}^{⊤}x_{j}\right)}^{2}\\ \;=\sum_{j=1}^{s_{1}}F_{1}(\hat{w})+\sum_{j=1}^{s_{2}}F_{2}(\hat{w})+\sum_{j=1}^{s_{3}}F_{3}(\hat{w})\\ ∴▽F(\hat{w})=\sum_{j=1}^{s_{1}}▽F_{1}(\hat{w})+\sum_{j=1}^{s_{2}}▽F_{2}(\hat{w})+\sum_{j=1}^{s_{3}}▽F_{3}(\hat{w}) \end{array}$$

From Equation (13), it should be easy to see that the aggregated gradient is just a sum of the gradients from each site. On the other hand, if the mean sum of squared errors is preferred i.e., $F\left(\hat{\text{w}}\right)=\frac{1}{m}\overset{m}{\underset{j=1}{\sum }}{\left(y_{j}-{\hat{\text{w}}}^{⊤}x_{j}\right)}^{2}$, which mathematically has the same minimizer as $\overset{m}{\underset{j=1}{\sum }}{\left(y_{j}-{\hat{\text{w}}}^{⊤}x_{j}\right)}^{2}$ since $F\left(\hat{\text{w}}\right)$ is convex, it can be shown that the aggregated gradient is a weighted average of the gradients from the local sites:

(14) $$\begin{array}{l} F(\hat{w})=\frac{1}{s_{1}+s_{2}+s_{3}}\sum_{j=1}^{s_{1}+s_{2}+s_{3}}F_{j}(\hat{w})\\ \;=\frac{1}{s_{1}+s_{2}+s_{3}}(\frac{s_{1}}{s_{1}}\sum_{j=1}^{s_{1}}F_{j}(\hat{w})+\frac{s_{2}}{s_{2}}\sum_{j=1}^{s_{2}}F_{j}(\hat{w})\\ \;+\frac{s_{3}}{s_{3}}\sum_{j=1}^{s_{3}}F_{j}(\hat{w}))\\ \;=\frac{1}{s_{1}+s_{2}+s_{3}}(s_{1}F_{1}(\hat{w})+s_{2}F_{2}(\hat{w})+s_{3}F_{3}(\hat{w}))\\ ∴▽F(\hat{w})=\frac{1}{s_{1}+s_{2}+s_{3}}(s_{1}▽F_{j}(\hat{w})+s_{2}▽F_{j}(\hat{w})+s_{3}▽F_{j}(\hat{w})) \end{array}$$

Algorithm 3 shows the steps involved in multi-shot regression. In order to update the parameters (*here*, $\hat{\text{w}}$), any off-the-shelf optimization scheme, for example, gradient descent, adagrad (Duchi et al., 2011), adadelta (Zeiler, 2012), momentum gradient descent (Rumelhart et al., 1986), nesterov accelerated gradient descent (Nesterov et al., 1983), Adam (Kingma and Ba, 2014) could have been used. The choice of scheme adopted could depend on the data being analyzed, Moreover, additional considerations have to be given to the stopping criterion tolerance, the number of iterations, the choice of learning rate and any other additional hyper-parameters depending on the scheme utilized. In some cases, the choice of optimization scheme can result in an analysis which could take minutes, days or years to arrive. In our tests, we found out that the Adam optimization scheme performs extremely well on the real dataset and hence has been adopted to perform the multi-shot regression.

#### 2.1.4. Other statistics

In addition to generating the weights of the covariates (regression parameters), one would also be interested in determining the overall model performance given by goodness-of-fit or the coefficient of determination (*R*^2^) as well as the statistical significance of each weight parameter (*t*-value or *p*-value).

As demonstrated in Algorithm 4 (Ming et al., 2017), determining *R*^2^ entails calculating the sum-square-of-errors (*SSE*) as well as total sum of squares (*SST*) which are evaluated at each local site and then aggregated at the global site to evaluate *R*^2^ given by 1−*SSE*/*SST*. An intermediary step before the calculation of *SST* is the calculation of the global $\bar{\text{y}}$ which is determined by taking a weighted average of the local ${\bar{\text{y}}}_{j}$ weighted on the size of data at each local site.

**Algorithm 4:**
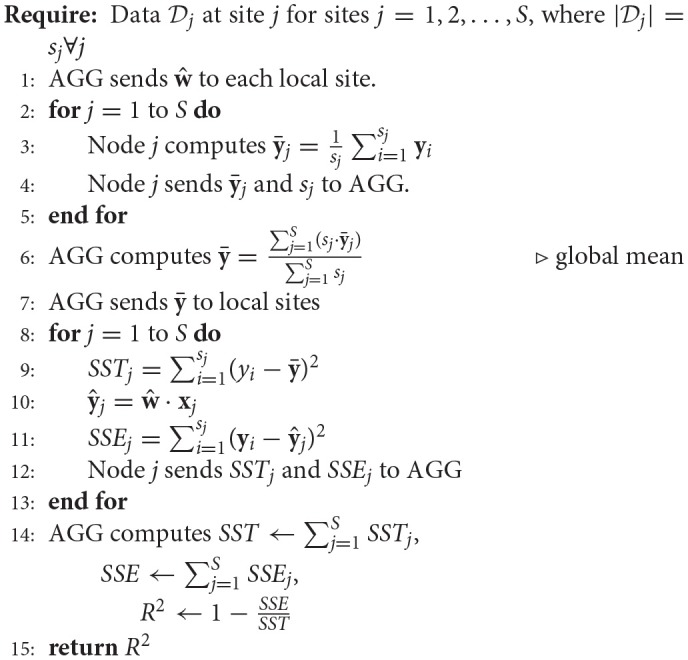
Decentralized *R*^2^ calculation

Algorithm 5 (Ming et al., 2017) details the steps involved in calculating the *t*-values (and therefore *p*-values) of each regression parameter. Assuming the weight vector has been calculated using either the single-shot or multi-shot regression, the global weight vector ($\hat{\text{w}}$) is sent to each of the local sites where the local covariance matrix as well as the sum-square-of-errors is calculated and sent back along with the data size to the aggregator (*AGG*) which then utilizes that information to calculate the *t*-values for each parameter (or coefficient). Once, the *t*-values have been calculated, the corresponding two-tailed *p*-values can be deduced using any publicly available *distributions* library.

**Algorithm 5:**
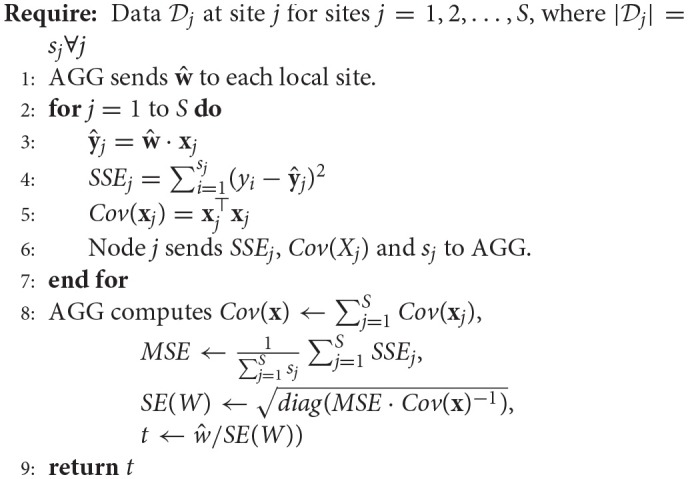
Decentralized *t*-value calculation

#### 2.1.5. Bandwidth and complexity

For singleshot regression, each site communicates a local weight vector ${\hat{\text{w}}}_{j}$ of size (*d* + 1) to the aggregator in addition to the cardinality of the dataset at each site $|D_{j}|=s_{j}$, a scalar. Once all the information is aggregated, a weighted average of the local ${\hat{\text{w}}}_{j}$s with the weights being *s*_*j*_ performed to get the global weight vector $\hat{\text{w}}$. Assuming *s*_*j*_ > *d* and that the normal equation is used to get the local weight vectors ${\hat{\text{w}}}_{j}$s, the computational complexity is $O\left(d^{2}s_{j}\right)$ whereas the computational complexity of calculating the weighted average at the *AGG* is O(d).

In the case of decentralized regression with normal equation, the first step (at each site) includes the calculation of **x**^⊤^**x** (at $O\left(d^{2}s_{j}\right)$) and **x**^⊤^**y** (at $O\left(ds_{j}\right)$) with an overall complexity of $O\left(d^{2}s_{j}\right)$. A total information of $\overset{S}{\underset{j=1}{\sum }}\left\{s_{j}\times \left[{\left(d+1\right)}^{2}+\left(d+1\right)\right]\right\}$ is communicated to the *AGG* where they are aggregated (as shown in Algorithm 2) to obtain the global weight vector $\hat{\text{w}}$ at $O\left(d^{3}\right)$.

Contrary to where the computation starts in the case of singleshot or DRNE, the computation/communication starts from the *AGG* in multishot regression. The *AGG* initializes the $\hat{\text{w}}$ and communicates the (*d* + 1)-sized vector to each of the *S* sites. At every iteration, each site *j* then calculates the gradient vector (O(d)) and sends it back to the *AGG* which again means the communication *S*×(*d* + 1) accounting for S sites. At the *AGG*, steps 7 though 12 (refer to Algorithm 3) are performed at an order of O(d) which are again sent back to each of the local sites, implying a communication of *S* × *d*, for the next iteration of the gradient descent.

The above treatment of communication bandwidth and complexity is subject to certain considerations *viz*., the number of covariates, the number of samples at each site, the optimization scheme used in the calculation of **x**^⊤^**x**, the stopping criterion, etc.

## 2.2. Decentralized DFNC

In this section, we briefly present our initial work toward performing dynamic functional network connectivity (dFNC) analysis in a decentralized framework. As mentioned earlier, dFNC is a multi-step pipeline finds common states in subject fMRI time-courses (TCs), and is often done by clustering a sliding window over subject time-courses, as is done (e.g., Allen et al., 2014; Damaraju et al., 2014). Thus, we present methods for decentralized spatial ICA along with decentralized K-Means clustering. Our presentation here is by no means a rigorous take on dFNC which we save for future work.

### 2.2.1. Decentralized group spatial ICA

Following preprocessing, the first step in the dFNC pipeline includes group ICA (Calhoun et al., 2001). Since we are dealing with fMRI data, suppose that we now have data **X** ∈ ℝ ^*d* × *N*^, where *d* is the voxel-space of the data (in brain voxels), and *N* is the total number of time-points across all subjects in the network. In linear spatial ICA, we model each individual subject as a mixture of *r* many statistically independent spatial maps, **A** ∈ ℝ ^*d* × *r*^, and their time-courses, $\text{S}\in {\mathbb{R}}^{r\times N_{i}}$, where *N*_*i*_ is the length of the time-course belonging to subject *i*. In the decentralized case, we can model the global data set **X** as the column-wise concatenation of *s* sites in the temporal dimension, where each site is modeled as a set of subjects concatenated in the temporal dimension:

$$\begin{array}{l} \text{X}=\left[{\text{A}}_{1}{\text{S}}_{1}\;{\text{A}}_{2}{\text{S}}_{2}\;\cdots \;{\text{A}}_{s}{\text{S}}_{s}\right]\in {\mathbb{R}}^{d\times N}. \end{array}$$

Our goal is to learn a global unmixing matrix, **W**, such that $\text{X}\text{W}\approx \hat{\text{A}}$, where $\hat{\text{A}}\in {\mathbb{R}}^{d\times r}$ is a set of unmixed spatially independent components. To this end, we perform a decentralized group independent component analysis (dgICA). Our method consists first of the two-stage GlobalPCA procedure utilized in Baker et al. (2015). In this procedure, each site first performs subject-specific LocalPCA dimension-reduction and whitening to a common *k* principal components in the temporal dimension. A decentralized, second stage, then produces a global set of *r* spatial eigenvectors, **V** ∈ ℝ ^*r* × *d*^. As outlined in Baker et al. (2015), this second stage has sites pass locally-reduced eigenvectors to other sites in a peer-to-peer scheme, where upon receiving a set of eigenvectors, a site then stacks them in the column dimension, and performs a further reduction of the stacked matrix, which is then passed to the next peer in the network. This process iterates until the global eigenvectors reach some aggregator (*AGG*), or otherwise terminal site in the network.

**Algorithm 6:**
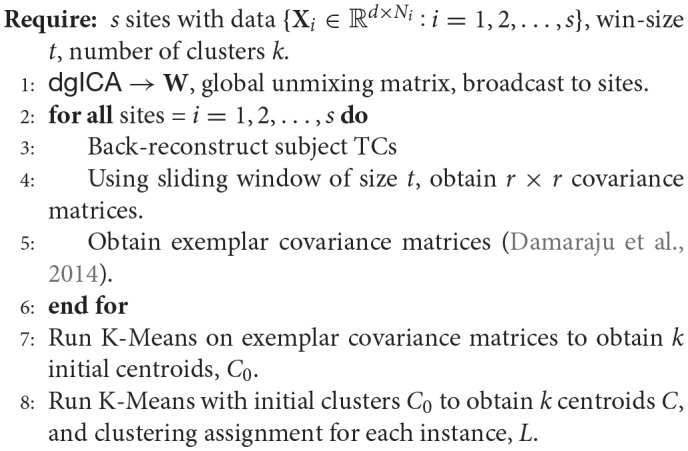
Decentralized group ICA algorithm (dgICA)

The aggregator site then performs whitening on these resulting eigenvectors, and runs a local ICA algorithm, such as infomax ICA (Bell and Sejnowski, 1995), to produce the spatial unmixing matrix, **W**. The global spatial eigenvectors, **V**, are then unmixed to produce $\hat{\text{A}}$ by computing $\hat{\text{A}}\approx \text{V}\text{W}$, which is shared across the decentralized network. Each site then uses this unmixing matrix to produce individual time-courses for each *i*-th subject by computing ${\text{A}}_{i}\approx {\text{X}}_{i}^{T}\text{S}$. Each site can then perform spatio-temporal regression back reconstruction approach (Calhoun et al., 2001; Erhardt et al., 2011) to produce subject-specific spatial maps.

### 2.2.2. Decentralized clustering

In order to perform dFNC in a decentralized paradigm, we first require a notion of decentralized clustering. Following the precedent of previous work in dFNC, we focus first on decentralized K-Means optimization, for which there exist a number of pre-established methods for decentralization. A number of methods utilize some manner of weighted centroid averaging, where each site in the network broadcasts updated centroids to an aggregator node which then computes the merged centroids, and rebroadcasts them to the local sites (Forman and Zhang, 2000; Dhillon and Modha, 2000; Jagannathan and Wright, 2005), though completely peer-to-peer approaches have also been proposed (Datta et al., 2006, 2009), as well as methods robust to asynchronous updates (Di Fatta et al., 2013). Though we have not found any methods which do this, methods which compute K-Means via gradient descent (Bottou, 2010) are also amenable to decentralization (Yuan et al., 2016). For simplicity's sake, we take the approach of centroid-averaging outlined in Dhillon and Modha (2000), and leave rigorous presentation and comparison of the remaining methods as future work.

**Algorithm 7:**
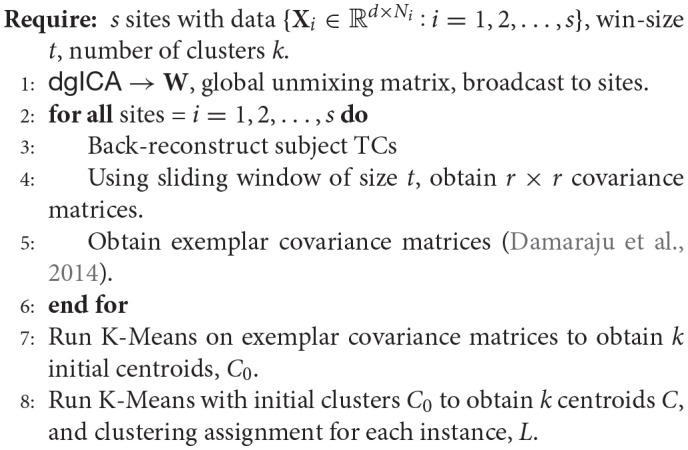
**Decentralized dFNC algorithm (**ddFNC**)**

To perform clustering for distributed dFNC, we first have each site separate its subjects into sliding-window time-courses, where the window length is fixed across the decentralized network. Additionally, initial clustering was performed on a subset of windows from each subject, corresponding to windows of maximal variability in correlation across component pairs. To obtain these exemplars, each site computes variance of dynamic connectivity across all pairs of components at each window. We then select windows corresponding to local maxima in this variance time-course. This resulted in an average of 8 exemplar windows per subject. We then perform decentralized K-Means on the exemplars to obtain a set of centroids, which are shared across the decentralized network, which we feed into a second stage of K-Means clustering.

For the second stage of decentralized clustering, at each iteration, each site computes updated centroids according to Dhillon and Modha (2000), which corresponds to a local K-Means update. These local centroids are then sent to the aggregator node, which computes the weighted average of these updated centroids, and re-broadcasts the updated global centroids until convergence.

### 2.2.3. Bandwidth and complexity

To compute the communication and complexity for ddFNC, we separately analyse the novel component algorithms of dgICA and dK-Means.

For decentralized group ICA, the communication of the algorithm is closely related to the communication of GlobalPCA. In the GlobalPCA algorithm given in Baker et al. (2015), each site communicates a *d* × *r* matrix of eigenvectors to the subsequent site until the aggregator is reached. After the aggregator performs ICA to obtain the global unmixing matrix, **W**, this matrix is broadcast to all other sites in the network. Thus, for a single, non-aggregator site, the total communication for dgICA is exactly *d* × *r* + *r*^2^. At the aggregator, the total communication is exactly *d* × *r* + *r*^2^ × *s* if the unmixing matrix is broadcast directly to each node. Of course, this cost could be mitigated by following a peer to peer communication scheme, and having other non-aggregator sites broadcast the unmixing matrix as well.

Next, we can compute the overall complexity of dgICA as the total complexity of local site operations. Consider an individual site, *i*, with *m* subjects, where the concatenated matrix is given as ${\text{X}}_{i}\in {\mathbb{R}}^{d\times N_{i}}$. In general, the complexity of SVD on the *N*_*i*_×*N*_*i*_ covariance matrix is $O\left(N_{i}^{3}\right)$, though this can be improved upon by using iterative methods, such as the MATLAB *svds* function. Thus, the complexity for the two-stage LocalPCA computation on one site is $O\left(2N_{i}^{3}\right)$. The per-site complexity for GlobalPCAis given as the complexity of a SVD computed on a *d* × *d* covariance matrix, which is created by concatenating the *k*_2_ eigenvectors from the previous site; i.e., the per-site complexity for GlobalPCA is $O\left(d^{3}\right)$. Finally, the complexity of ICA is exactly equal to the number of ICA iterations, J, which depends heavily on the choice of ICA algorithm, and hyper-parameter selection (see Bell and Sejnowski, 1995 for more details on the complexity of Infomax, for example). Thus, the total per-site complexity for dgICA is $O\left(N_{i}^{3}+d_{i}^{3}\right)$ for non-aggregator nodes, and $O\left(N_{i}^{3}+d_{i}^{3}+J\right)$ on the aggregator node. The overall runtime of dgICA is thus dependent on the computational resources available at each site, as well as the computational resources and ICA parameters chosen by the aggregator site.

Prior to performing K-Means, each site *i* computes *N*_*i, j*_−*w* windowed time-courses of length *w* on each subject *j*, computing the rank *r* covariance matrix for those windows. Thus, if there are *m*_*i*_ subjects at site *i*, the local complexity is $O\left(m_{i}\left(N-w\right)r^{3}\right)$ for this operation. No inter-site communication occurs during this process.

For decentralized K-Means, the communication between sites depends on the number of “K-Means Iterations,” J, i.e., the number of iterations required for the centroids to stabilize. J depends heavily on the initial centroids, the distance metric used, the distribution of the global data set, and other factors which make it difficult to compute exactly for arbitrary data. In each iteration of decentralized K-Means, we communicate *k* many centroids of size ℝ^*r*^^2^, for an average communication of $r^{2}\cdot k\cdot J$ from the sites to the aggregator. The aggregator, then, performs a total of $r^{2}\cdot k\cdot J\cdot s$ communication (Dhillon and Modha, 2000), which again, could be mitigated by passing centroids to intermediate sites, provided those sites can be trusted with the centroid information.

The time complexity of decentralized K-Means is described in Dhillon and Modha (2000). At each site, the distance and centroid recalculation computations come out to per-site complexity of $O\left(\left(3kr^{2}+M_{i}k+M_{i}r^{2}+kr^{2}\right)\cdot J\right)$ (Dhillon and Modha, 2000), where *M*_*i*_ is the number of instances at site *i*. The total number of computations consists of the sum of these site-wise complexities, and the centroid-averaging step with a complexity of $O\left(kr^{2}\right)$, for a total of $O\left(\left(3kr^{2}+Mk+Mr^{2}+kr^{2}\right)\cdot J\right)$, where *M* is the total number of data instances in the decentralized network.

Since dK-Means is computed twice for full ddFNC, once on the exemplars, and once on the global set of subject windows, the complete complexity of the clustering stage of the algorithm is given as the dK-Means complexity for *M* = ∑*E*_*i*_ added to the dK-Means complexity for *M* = ∑*m*_*i*_, i.e., $O\left(\left(3kr^{2}+\left(\sum E_{i}+\sum m_{i}\right)\left(k+r^{2}\right)+kr^{2}\right)\cdot J+kr^{2}\right)$.

The overall site-wise complexity and communication for ddFNC is just the sum of the site-wise communication and complexities for each of the stages described here. In the paradigm described here, the communication and complexity on the aggregator is generally more demanding than that on the individual sites, which makes sense for cases where the aggregator has sufficient and reliable network and hardware resources. In cases where this is not necessarily true, some of the aggregation tasks can be distributed to other sites in the network, thus reducing communication and complexity on the final aggregator. In the dgICA algorithm, performing ICA on the aggregator may become a bottleneck if the aggregator does not have sufficient computational resources to perform a standard run of ICA; however, this problem could be mitigated by performing a hardware check on sites in the consortium, and assigning the role of aggregator dynamically based on availability of computational resources. For more discussion of the particularities of network communication and other issues which may arise in decentralized frameworks like the one used for ddFNC, see Plis et al. (2016).

## 3. Data

### 3.1. Structural mri for decentralized VBM

As part of validating the proof-of-concept, we applied decentralized VBM to brain structure data collected on chronic schizophrenic patients and healthy controls. Specifically, the data comes from the Mind Clinical Imaging Consortium (MCIC) collection- a publicly accessible, on-line data repository containing curated anatomical and functional MRI, in addition to other data, collected from individuals with and without a schizophrenia spectrum disorder (Gollub et al., 2013) and available via the COINS data exchange https://coins.mrn.org (Scott et al., 2011).

Although more information about the MCIC can be found in Gollub et al. (2013), here we will report numbers for the final data used in this study as some subjects were excluded during the preprocessing phase. The final cohort for whom data are available includes 146 patients and 160 controls with site distribution as follows: Site B (IA) 40 patients/67 controls; Site D (MGH) 32/23; Site C (UMN) 32/26; Site A (UNM) 42/44, respectively. All subjects provided informed consent to participate in the study that was approved by the human research committees at each of the sites.

Briefly, T1-weighted structural MRI (sMRI) images were acquired with the following scan parameters: *TR* = 2, 530*ms* for 3 T, *TR* = 12*ms* for 1.5 T; *TE* = 3.79*ms* for 3 T, *TE* = 4.76*ms* for 1.5 T; *FA* = 7° for 3 T, *FA* = 20° for 1.5 T; *TI* = 1100*ms* for 3 T; *Bandwidth* = 181 for 3 T, *Bandwidth* = 110 for 1.5 T; *voxelsize* = 0.625 × 0.625*mm*; slice thickness 1.5 mm; *FOV* = 16−18*cm*.

The T1-weighted sMRI data were preprocessed using the Statistical Parametric Mapping software using unified segmentation (Ashburner and Friston, 2005), in which image registration, bias correction and tissue classification were performed using a single integrated algorithm resulting in individual brains segmented into gray matter, white matter and cerebrospinal fluid and nonlinearly warped to the Montreal Neurological Institute (MNI) standard space. The resulting gray matter concentration (GMC) images were re-sliced to 2 × 2 × 2*mm*, resulting in 91 × 109 × 91 voxels. Although one can obtain both modulated (Jacobian corrected) and unmodulated gray matter segmentations, in this study, we use unmodulated GMC maps to test our regression models.

To test the decentralized regression on the MCIC data described in the previous paragraph, we regress the age, diagnosis, gender and the site covariates on the voxel intensities (~600,000 voxels). All the decentralized computations discussed here have been performed on a single machine.

### 3.2. Functional MRI for dFNC

To evaluate ddFNC , we utilize imaging data from Damaraju et al. (2014) collected from 163 healthy controls (117 males, 46 females; mean age: 36.9 years) and 151 age- and gender matched patients with schizophrenia (114 males, 37 females; mean age: 37.8 years), for a total of 314 subjects.

The scans were collected during an eyes closed resting fMRI protocol at 7 different sites across United States and pass data quality control (see Supplementary Material). Informed and written consent was obtained from each participant prior to scanning in accordance with the Internal Review Boards of corresponding institutions (Keator et al., 2016). A total of 162 brain-volumes of echo planar imaging BOLD fMRI data were collected with a temporal resolution of 2 s on 3-Tesla scanners.

Imaging data for six of the seven sites was collected on a 3T Siemens Tim Trio System and on a 3T General Electric Discovery MR750 scanner at one site. Resting state fMRI scans were acquired using a standard gradient-echo echo planar imaging paradigm: FOV of 220 × 220 mm (64 × 64 matrix), TR = 2 s, TE = 30 ms, FA = 770, 162 volumes, 32 sequential ascending axial slices of 4 mm thickness and 1 mm skip. Subjects had their eyes closed during the resting state scan. Data preprocessing for dgICA was performed according to the preprocessing steps in Damaraju et al. (2014).

### 3.3. ddFNC experimental parameters

We verify that ddFNC can generate sensible dFNC clusters by replicating the centroids produced in Damaraju et al. (2014). We run both pooled and decentralized versions of our algorithm, and compare our results directly with the results provided by the authors of Damaraju et al. (2014). We thus closely follow the experimental procedure in Damaraju et al. (2014), with some of the additional post-processing omitted for simplicity. To evaluate the success of our pipeline, we run a simple experiment where we implement the ddFNC pipeline end-to-end on the data, simulating 314 subjects being evenly shared over 2 decentralized sites.

We set a window-length of 22 time-points (44 s), for a total of 140 windows per subject. For dgICA, we first estimate 120 subject-specific principal components locally, and reduce each subject to 120 points in the temporal dimension. Subjects are then concatenated temporally on each site, and we use the GlobalPCA algorithm in Baker et al. (2015) to estimate 100 spatial components, and perform whitening. We then use local infomax ICA (Bell and Sejnowski, 1995) on the aggregator to estimate the unmixing matrix **W**, and estimate 100 spatially independent components, $\hat{\text{A}}$. We then broadcast $\hat{\text{A}}$ back to the local sites, and each site computes subject-specific time-courses.

After spatial ICA, we have each site perform a set of additional post-processing steps prior to decentralized dFNC. First, we select 47 components from the initial 100, by computing components which are most highly correlated with the components from Damaraju et al. (2014). We then have each site drop the first 2 points from each subject, regress subject head movement parameters with 6 rigid body estimates, their derivatives and squares (total of 24 parameters). Additionally, any spikes identified are interpolated using 3rd order spline fits to good neighboring data, where spikes are defined as any points exceeding mean (FD) + 2.5 *std(FD) , where FD is framewise displacement [interpolating 0 to 9 points (mean, sd: 3, 1.76)].

For clustering, we forgo a separate elbow-criterion estimation, and use the optimal number of clusters from Damaraju et al. (2014), setting *k* = 5. For the exemplar stage of clustering, we evaluate 200 runs where we initialize centroids uniformly randomly from local data, and then run dK-Means using the cluster averaging strategy in Dhillon and Modha (2000). For our distance measure, we use scikit-learn (Pedregosa et al., 2011) to compute the correlation distance between covariance matrices following the methods in Damaraju et al. (2014). To keep our implementation simple, unlike Damaraju et al. (2014), we do not utilize graphical LASSO to estimate the covariance matrix, and thus do not optimize for any regularization parameters. Additionally, we do not perform additional Fisher-Z transformations or perform additional regularization using a previously computed static dFNC result. Future implementations may also utilize a decentralized static functional network connectivity (sFNC) algorithm as preprocessing, as is done for the pooled case in Damaraju et al. (2014). Finally, for the second stage of dK-Means, we initialize using the centroids from the run with the highest silhouette score, computed using the scikit-learn python toolbox (Pedregosa et al., 2011), again running dK-Means to convergence. After computing the centroids, we use the correlation distance and the Hungarian matching algorithm (Kuhn, 1955) to match both plotted spatial components from dgICA and the resulting centroids from dK-Means.

## 4. Results

### 4.1. Decentralized VBM results

**Figure 1:**
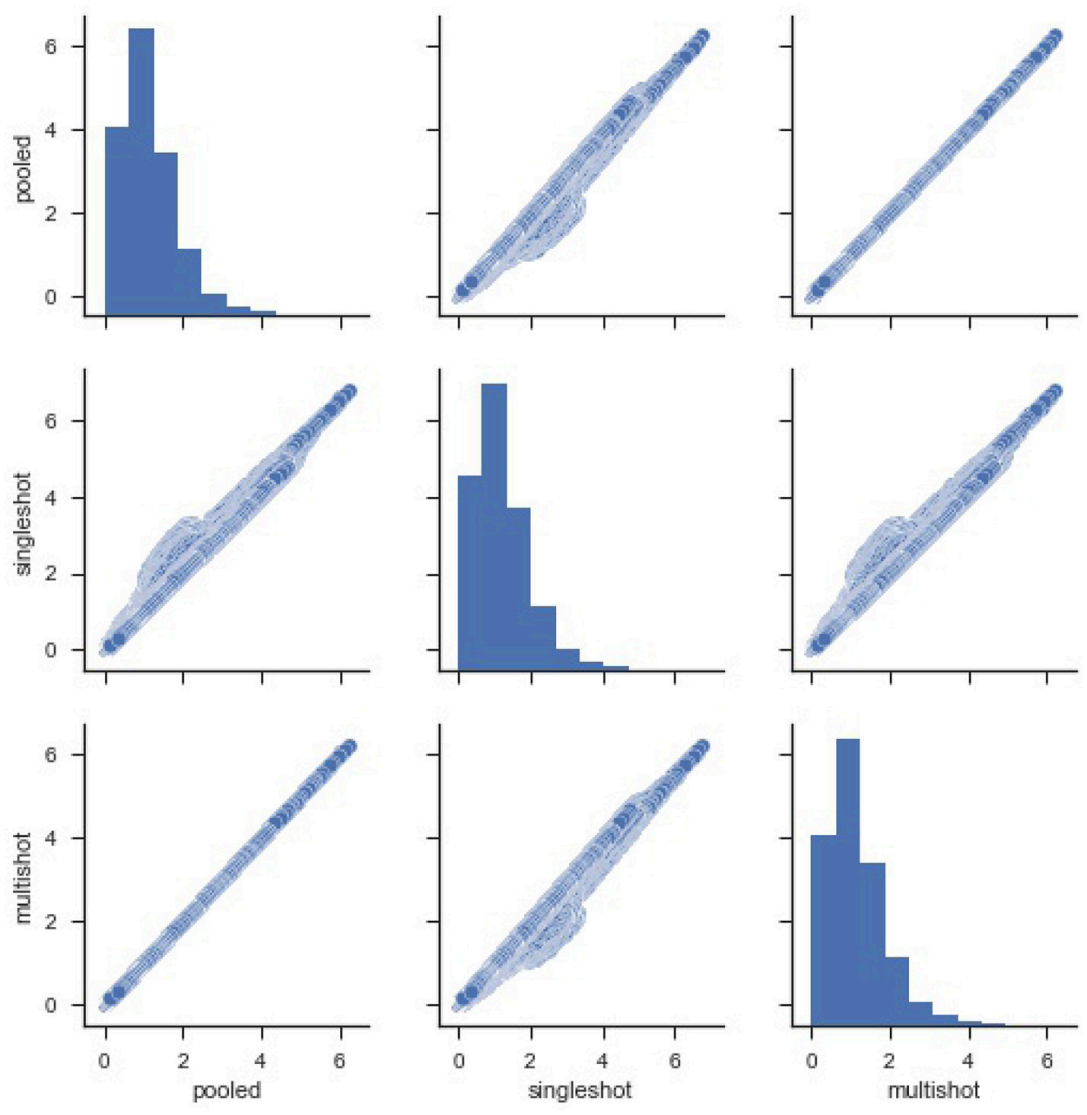
Pairwise plot of Sum Square of Errors (SSE) from pooled, single-shot and multi-shot regression. Although the distribution plot looks similar across the three regressions, the pooled regression vs. multi-shot regression scatter plot demonstrates how identical they are to each other.The scatter plot of pooled regression vs. single-shot regression demonstrates that the SSE values obtained from singles-shot regression are on the higher side compared to the values from pooled regression.

For starters, in order to compare the efficacy of each regression (single-shot and multi-shot) against the pooled case, we present a simple pairwise plot of the *SSE* of the regression performed on every voxel, Figure 1. In mathematical terms, the *SSE* represents lowest objective function value that could be attained from the regression model. It can be seen from Figure 1 that the *SSE* from multi-shot and pooled/centralized regression lie perfectly along a diagonal indicating the parameters obtained from them are identical. This can also be verified from Table 1 showing the correlation between the different *SSE*s. Please note that results from the decentralized regression with normal equation were not presented as it has been mathematically shown to be equivalent to that of a pooled regression.

**Table 1:** Correlation between *SSE* from pooled, single-shot and multi-shot regression.

	**Pooled**	**Single-shot**	**Multi-shot**
Pooled	1.000000	0.992905	1.000000
Single-shot	0.992905	1.000000	0.992905
Multi-shot	1.000000	0.992905	1.000000

It can be seen that the correlation between *SSE* from the centralized regression and multi-shot is 1. On the other hand, it can also be noticed that the *SSE* correlations between single-shot and pooled or single-shot and multi-shot are slightly lower than perfect correlation. The single-shot approach can be considered to be similar to a meta-analysis, whereas the multi-shot approach is basically a mega-analysis (i.e., equivalent to the pooled analysis).

**Figure 2:**
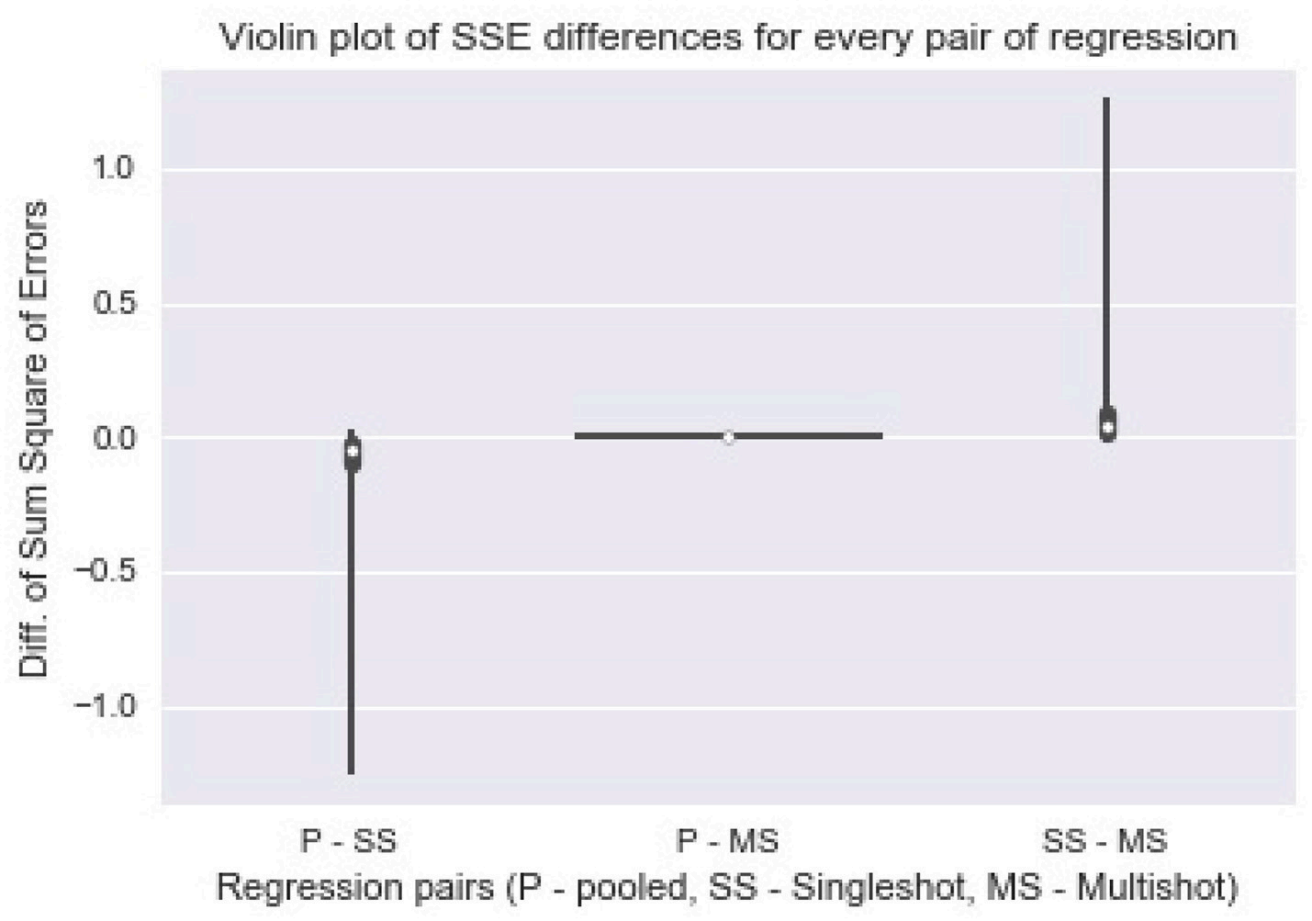
Violin plot of Sum Square of Error differences between every pair of regression. The plot of differences in SSE from pooled regression and multi-shot regression (P-MS) centered around 0 demonstrates how identical the results from the two regressions are. On the other hand, the SSE values from single-shot regression are higher compared to those from the pooled regression.

**Figure 3:**
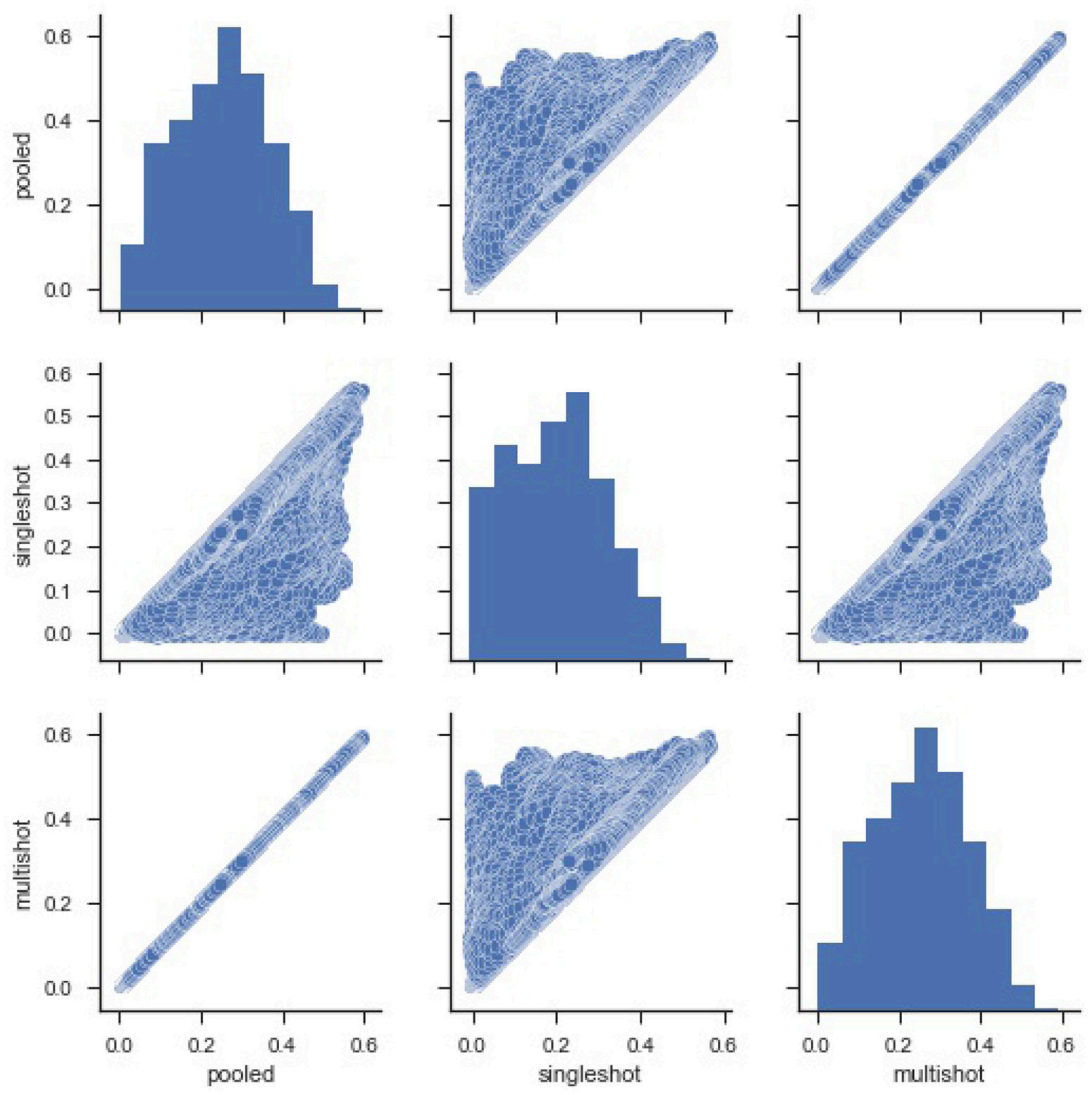
Pairwise scatter plots of Coefficient of Determination *R*^2^ from the three types of regression. It can be seen again that the *R*^2^ values for the regressions from multi-shot regression and pooled regression are exactly equal. The *R*^2^ values from single-shot regression are less than their corresponding values from pooled regression or multi-shot regression because the model being fit in single-shot has fewer covariates (Note, one of the limitations of the single-shot is that the site specific covariates could not be included as it introduces collinearity).

Figure 2 shows a violin (distribution) plot of the difference in *SSE* from every pair of regression. Evidently, the differences in *SSE* between pooled and multi-shot regression are centered around 0. To reinforce our notion that the multi-shot is superior to single-shot we take a look at the *R*^2^ values from the different regressions and compare. It can be seen from Figure 3 that the *R*^2^ values from multi-shot and pooled regression align perfectly along a diagonal (correlation = 1, refer to Table 2) or have exactly the same distribution, whereas those from single-shot are all over the place.

**Table 2:** Correlation between *R*^2^ from pooled, single-shot and multi-shot regression.

	**Pooled**	**Single-shot**	**Multi-shot**
Pooled	1.000000	0.906662	1.000000
Single-shot	0.906662	1.000000	0.906662
Multi-shot	1.000000	0.906662	1.000000

**Figure 4:**
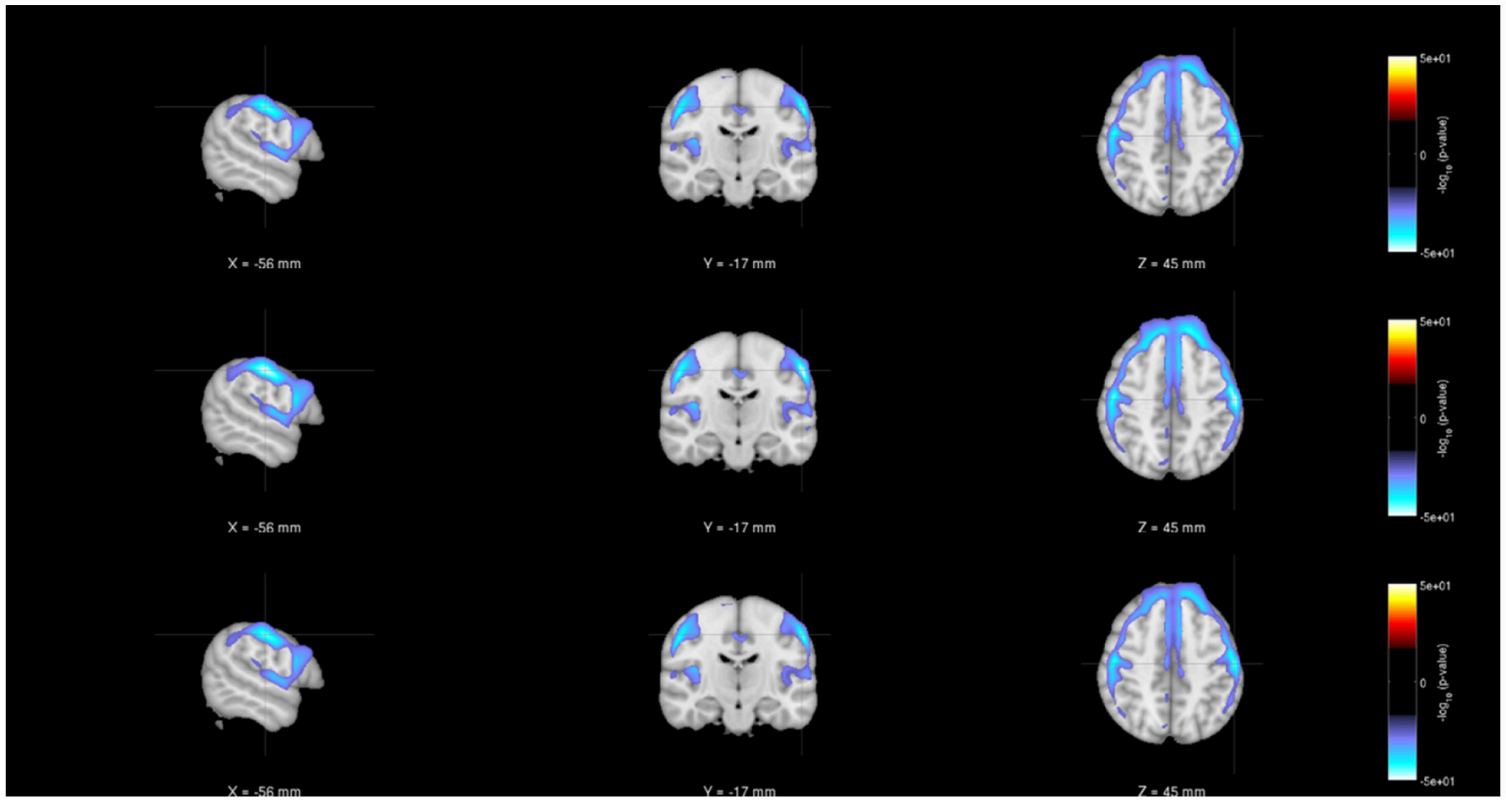
Rendered images of voxel-wise significance values (−*log*_10_*p*-value × sign(*t*)) for the covariate “Age” from pooled regression **(Top)** and single-shot regression **(Center)**, and multi-shot regression **(Bottom)** overlaid on MNI average template. One could see that the regions with expected gray matter decrease as age increases are similar from all kinds of regression. Although the single-shot regression uses fewer covariates, the similarity of the rendered images with those of pooled regression or multi-shot regression indicate the relative weight or orientation of the corresponding β coefficient will be similar to those from pooled/multi-shot regression.

**Figure 5:**
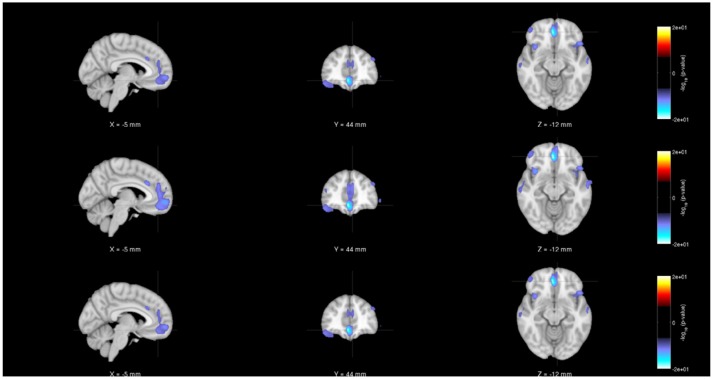
Rendered images of voxel-wise significance values (−*log*_10_*p*-value × sign(*t*)) for the covariate “Diagnosis” from pooled regression **(Top)** and single-shot regression **(Center)** and multi-shot regression **(Bottom)** overlaid on MNI average template. Regardless of the type of regression performed, the images indicate that in the medial frontal and bilateral temporal lobe/insula there is a significant gray matter density reduction for schizophrenic patients compared to the same regions of the healthy subjects.

**Figure 6:**
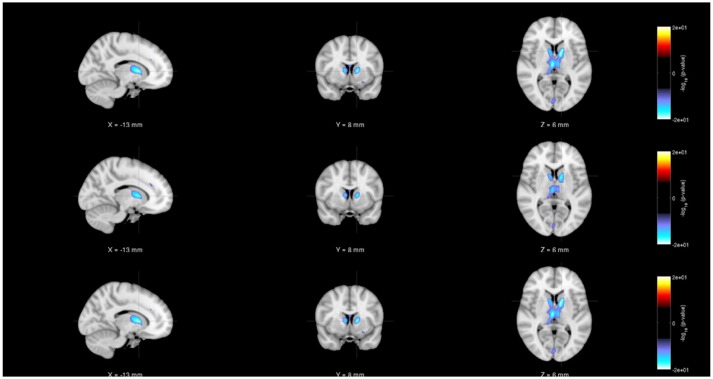
Rendered images of voxel-wise significance values (−*log*_10_*p*-value × sign(*t*)) for the covariate “Gender” from pooled regression **(Top)** and single-shot regression **(Center)** and multi-shot regression **(Bottom)** overlaid on MNI average template. It can be seen from all the three rendered images that there is a significant amount of gray matter reduction in the sub-cortical regions for males. Since we are using unmodulated gray matter maps, these sex differences could be due to changes in brain volumes.

As noted earlier, in addition to evaluating the regression model parameters, researchers will also be interested in understanding the statistical significance of the various parameter estimates. Figures 4–6 show the statistical significance of each covariate (age, diagnosis and gender), from both centralized and decentralized regressions performed against each voxel, plotted on an MNI brain template. Figure 4 shows the brain images with the −(*log*_10_*p*-val × sign(*t*))-values for the weight parameter corresponding to “Age.” It is notable to see that the results from the multi-shot regression have a perfect correlation to those from the pooled version. Moreover, the observations show the expected decrease in gray matter concentration as age increases. Figures 5, 6 show the rendered images for −*log*_10_*p*-values for the “Diagnosis” and “Gender” covariate, respectively.

### 4.2. dDFNC results

**Figure 7:**
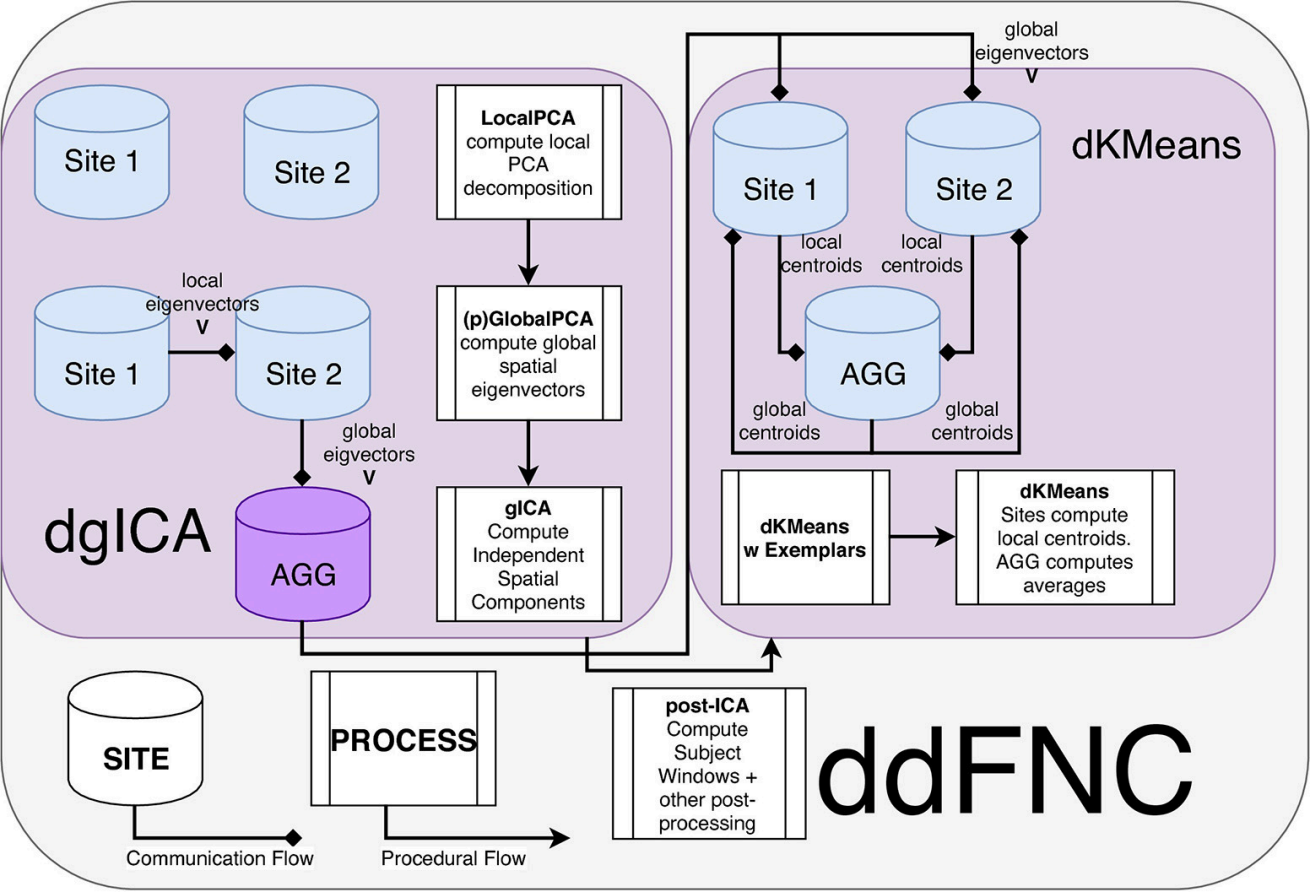
Flowchart of the ddFNC procedure e.g., with 2 sites. To perform dgICA, sites first locally compute subject-specific LocalPCA to reduce the temporal dimension, and then use the GlobalPCA procedure from Baker et al. (2015) to compute global spatial eigenvectors, which are then sent to the aggregator. The aggregator then performs ICA on the global spatial eigenvectors, using InfoMax ICA (Bell and Sejnowski, 1995) for example, and passes the resulting spatial components back to local sites. The dK-Means procedure then iteratively computes global centroids using the procedure outlined in Dhillon and Modha (2000), first computing centroids from subject exemplar dFNC windows, and then using these centroids to initialize clustering over all subject windows.

**Figure 8:**
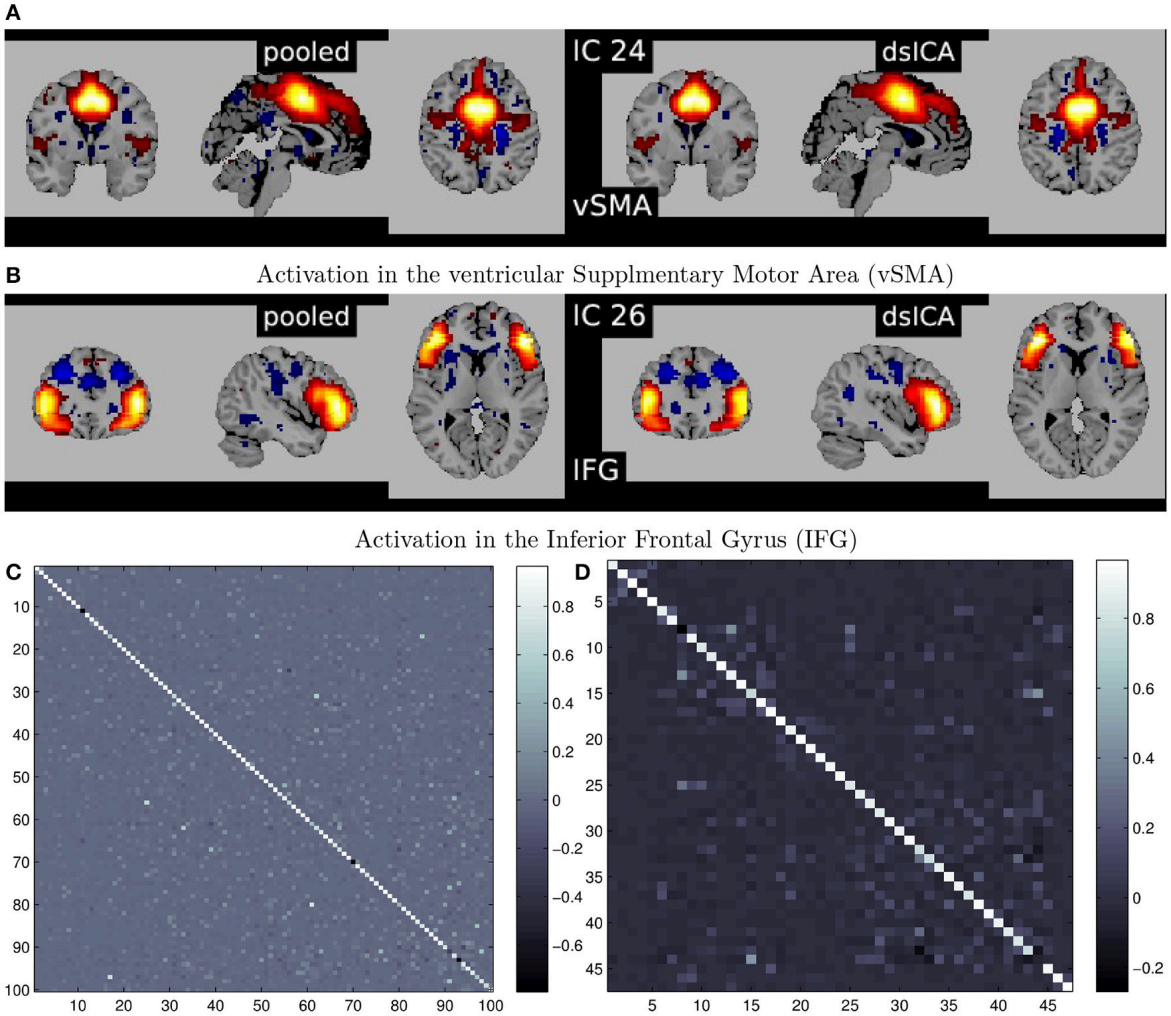
**(A,B)** Illustrate examples of matched spatial maps from dgICA and pooled ICA. **(C,D)** Show the correlation of the components between pooled spatial ICA and dgICA after hungarian matching. **(C)** Shows correlation between all 100 components, and **(D)** Shows correlation between the 47 neurological components selected in Damaraju et al. (2014).

A summary of the complete steps in the decentralized dFNC pipeline is given in Figure 7. In Figure 8, we plot some examples of the components estimated from decentralized spatial ICA in comparison with the spatial components from Damaraju et al. (2014), after performing Hungarian matching between the estimated spatial maps. We also plot the correlation of the components from our ICA implementation in comparison to the components from Damaraju et al. (2014). Indeed, the estimated components are highly correlated with the results from Damaraju et al. (2014), for all 100 estimated components, as well for the 47 selected neurological components from Damaraju et al. (2014), indicating that dgICA is able to produce results comparable to the pooled case. We include additional spatial maps for all 47 estimated spatial components in the Supplementary Material.

**Figure 9:**
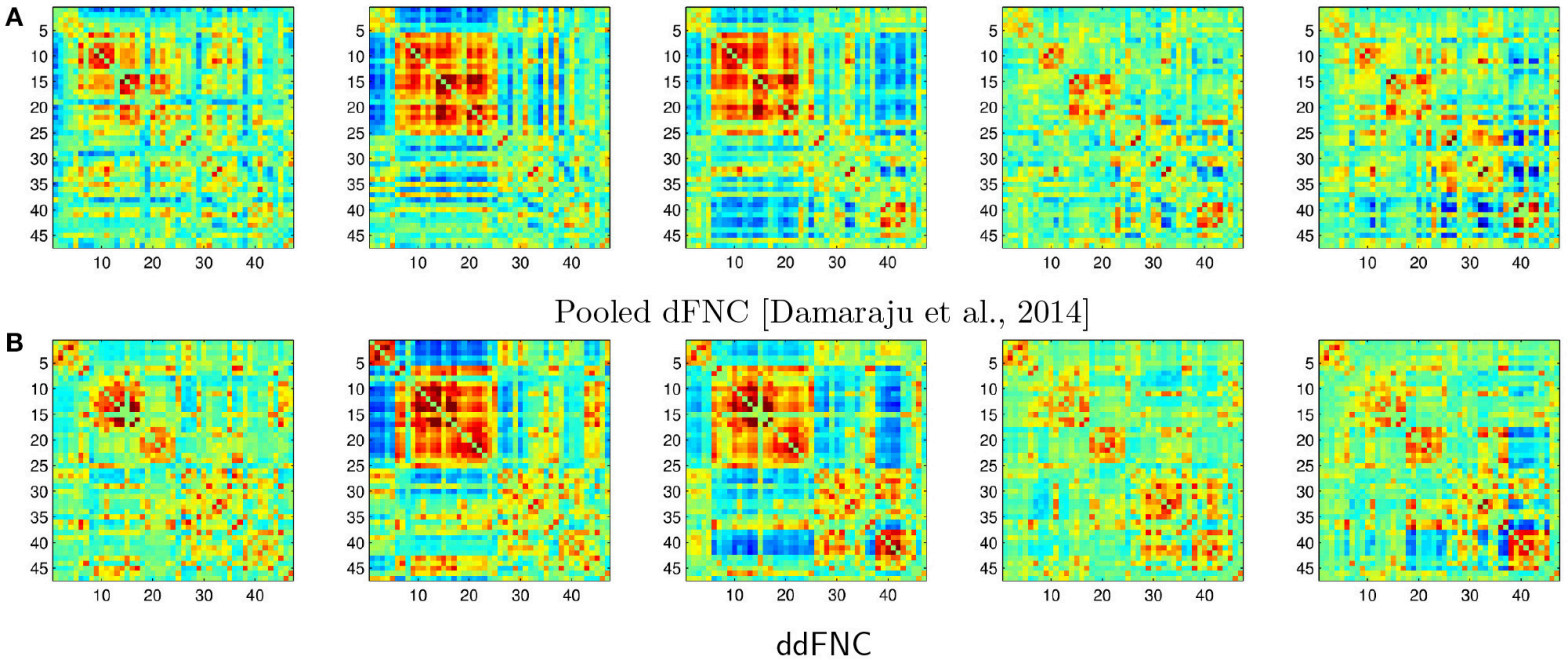
The *k* = 5 centroids for pooled dFNC from Damaraju et al. (2014) **(A)**, and the hungarian-matched centroids from ddFNC **(B)**.

In Figure 9, we plot the centroids from Damaraju et al. (2014) (Figure 9A), as well as the centroids estimated using decentralized dFNC (Figure 9B). Indeed, the centroids found using ddFNC prove similar to the centroids found in Damaraju et al. (2014), with centroids 2 and 3 being the closest matches under correlation distance.

## 5. Discussion

The results described in the previous section demonstrate the fidelity of decentralized regression and decentralized dynamic function network connectivity in analyzing neuroimaging data.

Although single-shot regression is simple and easy to implement, it limits our ability to incorporate site covariates and thus might not be extremely helpful. The decentralized regression with normal equation and multi-shot regression are superior to single-shot regression because not only do they allow incorporating site related variables but also give exact results as the pooled regression. The linearity and convexity of the regression objective function made this possible and thus are an excellent alternative to perform regression on multi-site datasets.

In terms of the regression objective function, either the sum of squared errors or mean sum of squared errors can be used in practice. However, it's mathematically convenient to use sum of squared errors which subsequently entails (at the *AGG*) a simple addition of the gradients (O(1)) instead of a weighted average of the gradients (O(n)). Added to that, we also showed how the sample size at the local sites has no bearing on the final results.

On a more practical note, the need for multi-shot regression might not arise often in a neuroimaging setting where the number of covariates is usally small. In such cases, the decentralized regression with normal equation will suffice. However, in decentralized settings where the number of covariates is usually large (machine learning/big data) the multi-shot regression comes to the fore. From a computational time standpoint, and as discussed in the computational complexity section, it should be obvious that the multi-shot regression takes more time to complete than the decentralized regression with normal equation as it involves iteratively passing the gradients between the local nodes and the *AGG*. It is worth mentioning that although the decentralized regression algorithms demonstrated here pertain to a simple linear regression model, these algorithms can easily be extended to more complex models with polynomial terms or interaction terms as well as to ridge regression, lasso regression, and elastic net regression.

Regarding ddFNC, we plan on performing a more robust analysis, going into the future, as a stand-alone algorithm, particularly with respect to different variations on the dK-Means optimization and initialization, or with differing versions of ICA on the aggregator (*AGG*) node, such as fastICA (Koldovský et al., 2006), Entropy Bound Minimization (Li and Adali, 2010), and others. Additionally, the possibility of performing a decentralized static FNC either as a preprocessing step to ddFNC or a separate analysis is attractive. One other avenue worth exploring with ddFNC is the flow of information across the decentralized network. In particular, since the GlobalPCA step in dgICA already makes the procedure partially peer-to-peer, it makes sense to explore adding this functionality to the dK-Means methods to preserve this peer-to-peer structure. Finally, we plan to evaluate privacy-sensitive versions of ddFNC, utilizing differential-privacy or other privacy measures as a way to perform these analyses with some assurance of per-subject privacy in the decentralized network.

Finally, we note that the decentralization of algorithms in a neuroimaging setting emphasizes the importance of analysis on data present at multiple sites, the decentralization discussed herewith is no different from other decentralized algorithms discussed elsewhere in literature. The *AGG* is not really a master node *per se* but in fact one of the local sites itself. The term *AGG* was introduced to separate all the other local sites from that site where the results are accumulated.

## 6. Conclusion

In this paper, we presented a simple case study of how voxel-based morphometry and dynamic functional network connectivity analysis can be performed on multi-site data without the need for pooling data at a central site. The study shows that both the decentralized voxel-based morphometry as well as the decentralized dynamic functional network connectivity yield results that are comparable to its pooled counterparts guaranteeing a virtual pooled analysis effect by a chain of computation and communication process. Other advantages of such a decentralized platform include data privacy and support for large data. In conclusion, the results presented here strongly encourage the use of decentralized algorithms in large neuroimaging studies over systems that are optimized for large-scale centralized data processing.

## Ethics statement

For the MCIC data, all subjects provided informed consent to participate in the study that was approved by the human research committees at each of the sites (UNM HRRC #03-429; UMinn IRB #0404M59124; MGH IRB# 2004P001360; UIowa IRB #1998010017). In addition to the informed consent, all patients successfully completed a questionnaire verifying that they understood the study procedures.

For fBIRN data, all subjects provided informed consent to participate in the study that was approved by the human research committees of each of the participating institutes in the fBIRN data repository.

## Author contributions

HG implemented the decentralized regression algorithms on structural MRI data and wrote the regression part of the paper. BB implemented the decentralized dynamic functional network connectivity pipeline on functional MRI data and wrote that part of the paper. ED contributed immensely to the analysis as well as interpretation of the results from both decentralized regression and decentralized dFNC pipeline. SRP contributed to the brain imaging data preprocessing pipeline. SMP proposed the decentralized data analysis system and led the algorithm development effort. RS helped formulate the decentralized regression with normal equation and development of decentralized spatial ICA. VC led the team and formed the vision.

### Conflict of interest statement

The authors declare that the research was conducted in the absence of any commercial or financial relationships that could be construed as a potential conflict of interest.
