# Supplementary material for: Decentralized Analysis of Brain Imaging Data: Voxel-Based Morphometry and Dynamic Functional Network Connectivity
Source: Front Neuroinform. 2018 Aug 27;12:55. doi: 10.3389/fninf.2018.00055 (PMC6119966; doi:10.3389/fninf.2018.00055)
Supplement: Supplementary file 1 [file Data_Sheet_1.PDF]

Supplementary Material:
Decentralized Analysis of Brain Imaging Data: Examples from structural and functional MRI

Gazula, H., Baker, B., Damaraju, E., Plis, S., Panta, S., Silva, R. and Calhoun, V.

*Correspondence:
Author Name: Harshvardhan Gazula
hgazula@mrn.org

1 SUPPLEMENTARY DATA

No supplementary data was provided

2 SUPPLEMENTARY TABLES AND FIGURES

To demonstrate the fact that there weren't significant differences in the weight vector for pooled and multi-shot regression, we also plotted the weight parameter with respect to each type of regression as well the differences for every pair of regression. For instance, Figures S1 and S2 show the brain images with the weight parameter corresponding to the 'Age' covariate. No significant differences can be found between the pooled and multishot case. Similarly, Figures S3, S4 and Figures S5, S6 and represent the weight parameter and weight parameter differences for the 'Diagnosis' and 'Gender' covariate respectively.

2.1 Figures

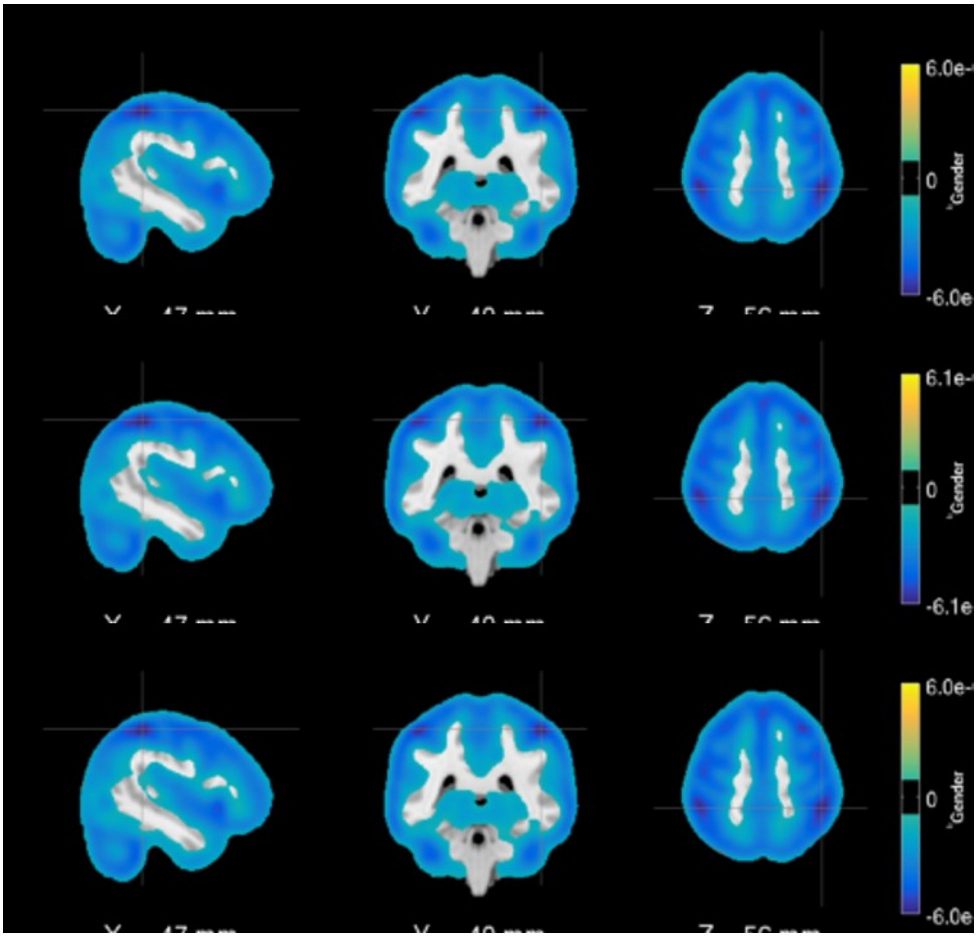

Figure S1. Comparison of β_{Age} for each voxel from pooled regression (top), singe-shot regression (center), and multi-shot regression (bottom)

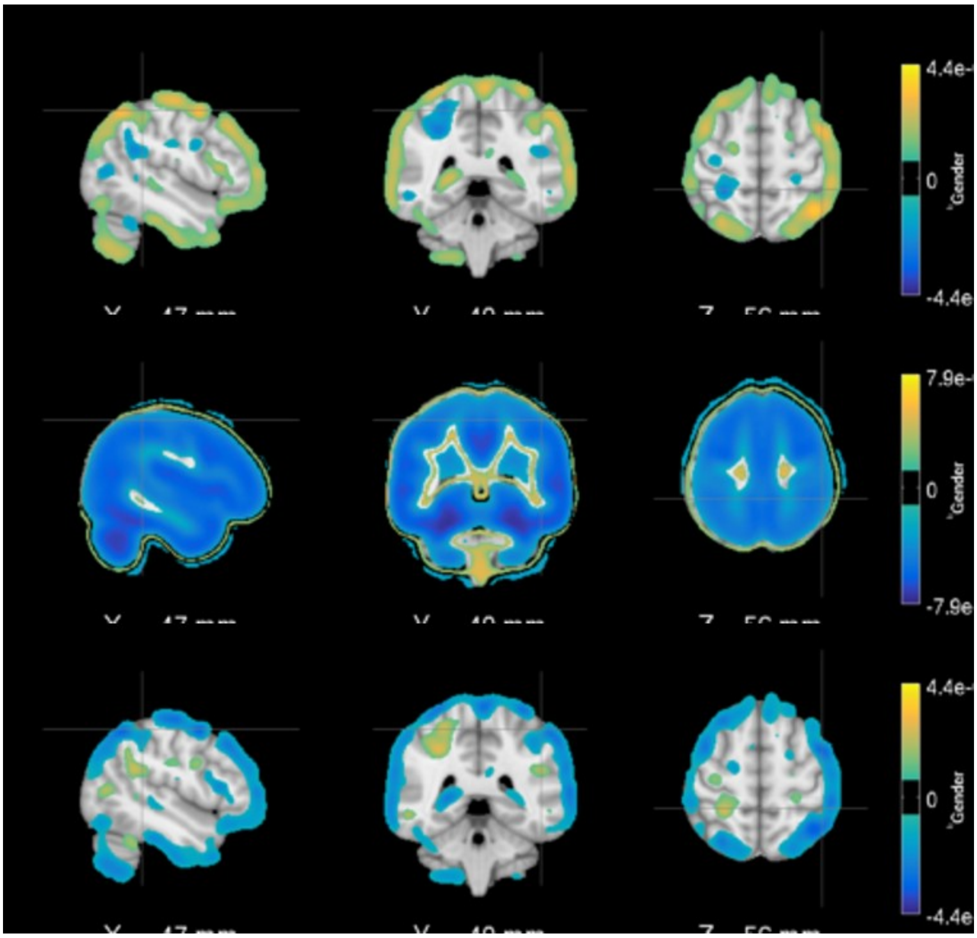

Figure S2. Plot of Difference in β_{Age} for each voxel. $\beta_{Age,pooled} - \beta_{Age,singleshot}$(top), $\beta_{Age,pooled} - \beta_{Age,multishot}$(center), and $\beta_{Age,singleshot} - \beta_{Age,multishot}$(bottom)

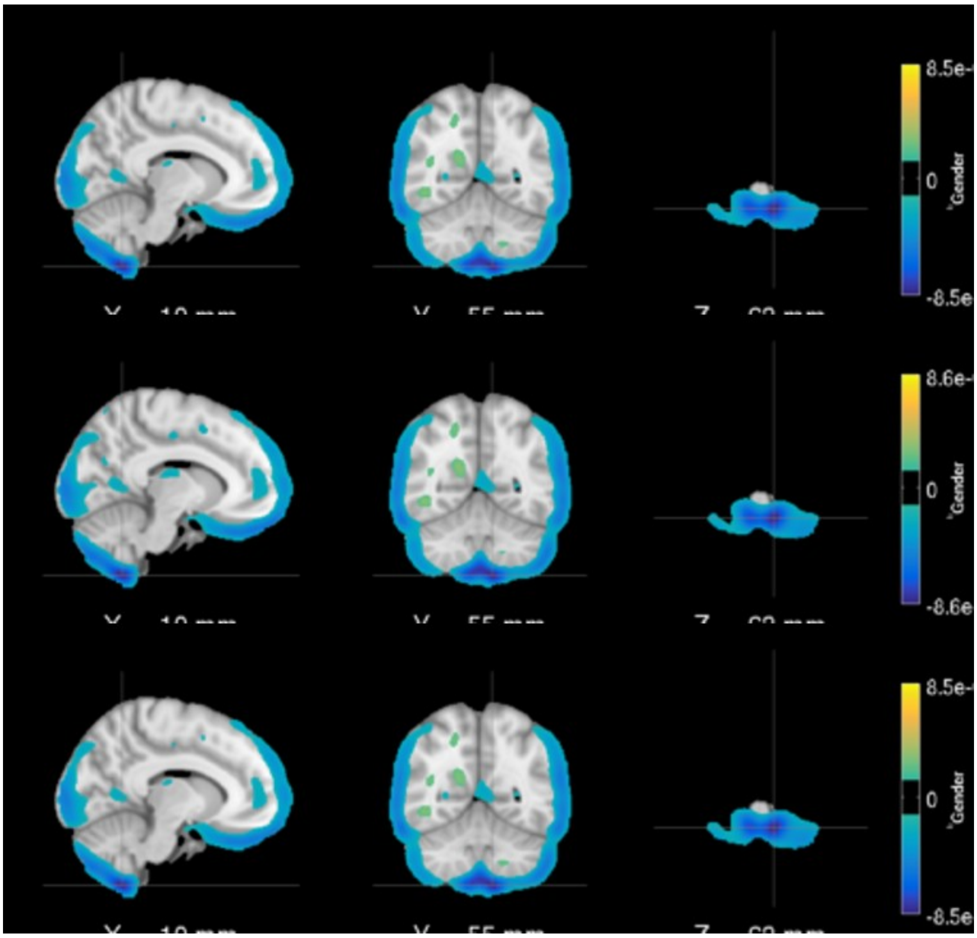

Figure S3. Comparison of $\beta_{Diagnosis}$ for each voxel from pooled regression (top), singe-shot regression (center), and multi-shot regression (bottom)

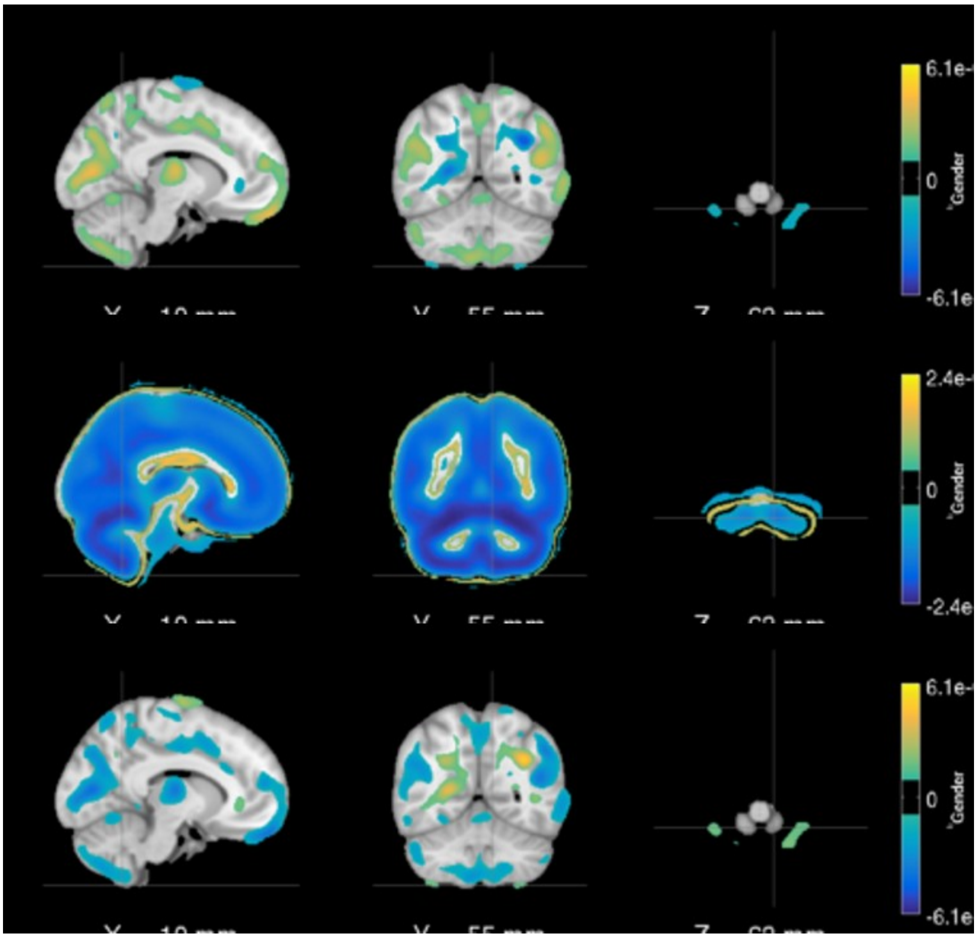

Figure S4. Plot of Difference in $\beta_{Diagnosis}$ for each voxel. $\beta_{Diagnosis,pooled} - \beta_{Diagnosis,singleshot}$(top), $\beta_{Diagnosis,pooled} - \beta_{Diagnosis,multishot}$(center), and $\beta_{Diagnosis,singleshot} - \beta_{Diagnosis,multishot}$(bottom)

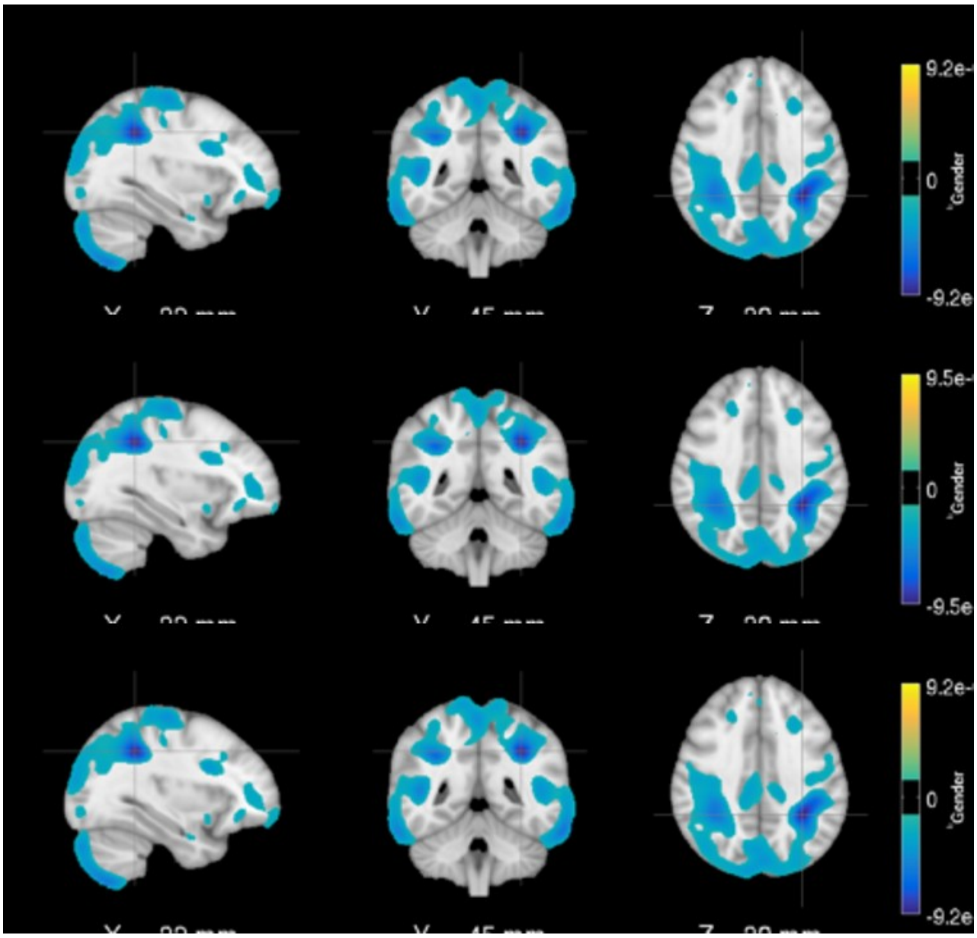

Figure S5. Comparison of β_{Gender} for each voxel from pooled regression (top), singe-shot regression (center), and multi-shot regression (bottom)

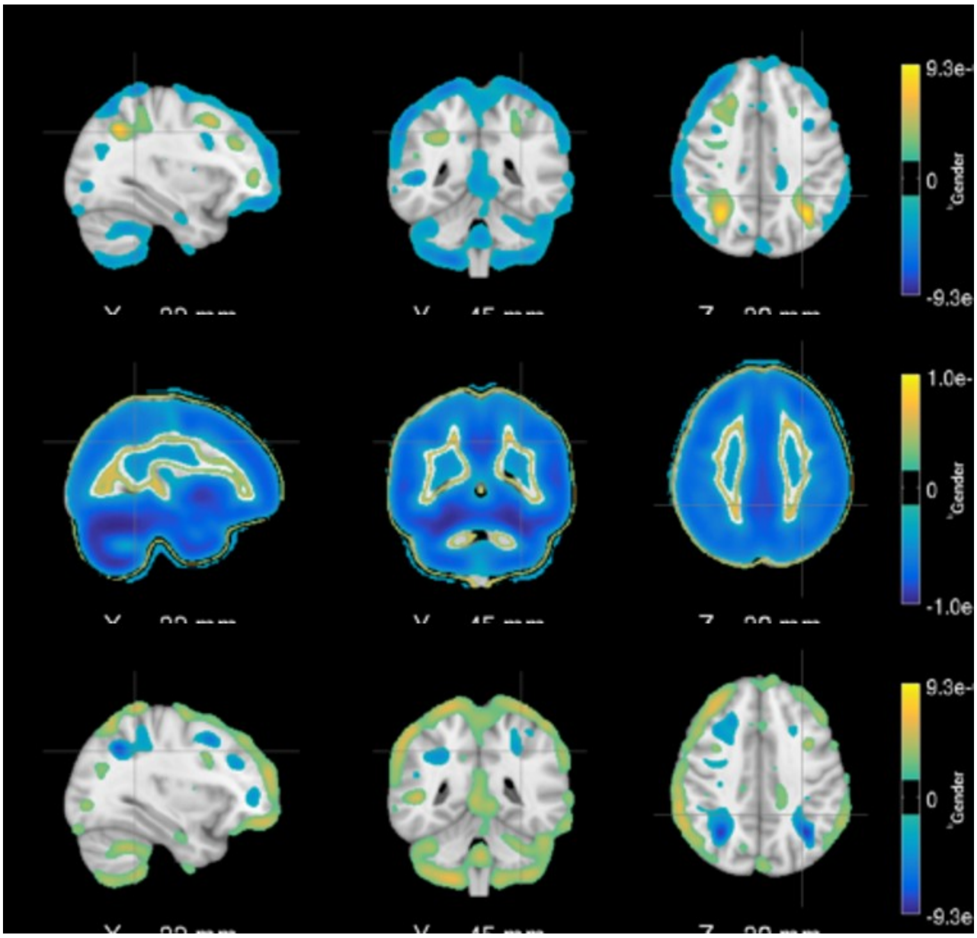

Figure S6. Plot of Difference in β_{Gender} for each voxel. $\beta_{Gender,pooled} - \beta_{Gender,singleshot}$(top), $\beta_{Gender,pooled} - \beta_{Gender,multishot}$(center), and $\beta_{Gender,singleshot} - \beta_{Gender,multishot}$(bottom)

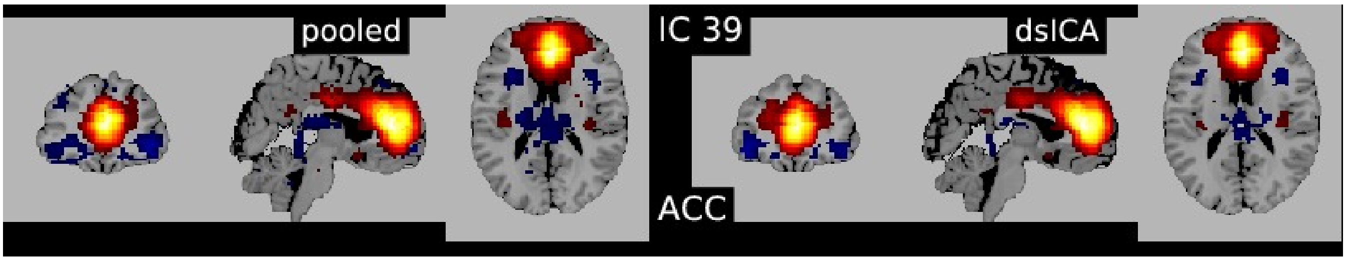

Figure S7. Activation in the ACC region of the brain, estimated by pooled ICA and dgICA

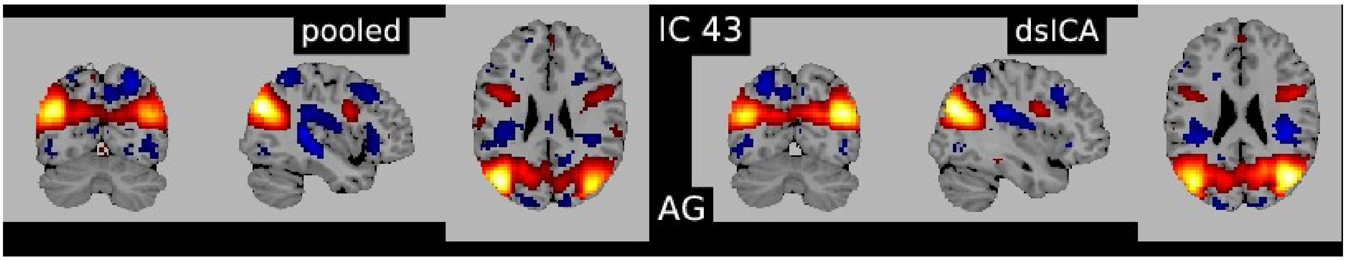

Figure S8. Activation in the AG region of the brain, estimated by pooled ICA and dgICA

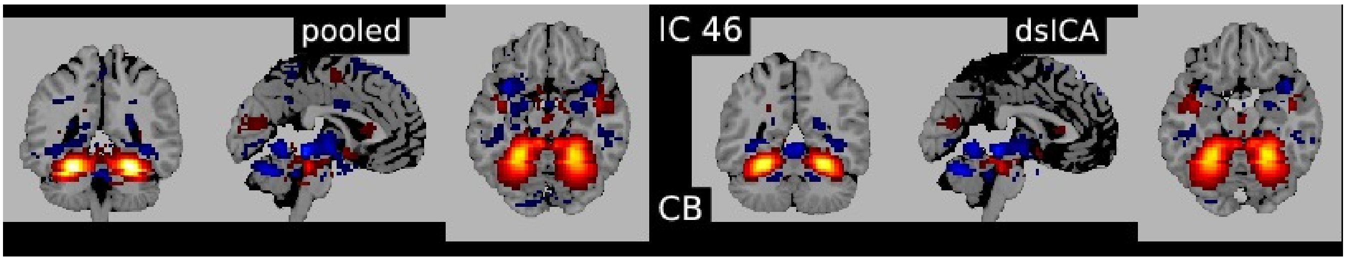

Figure S9. Activation in the CB region of the brain, estimated by pooled ICA and dgICA

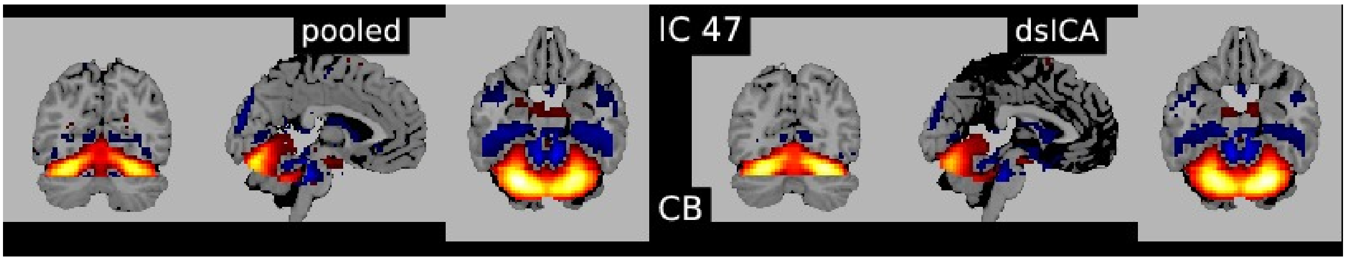

Figure S10. Activation in the CB region of the brain, estimated by pooled ICA and dgICA

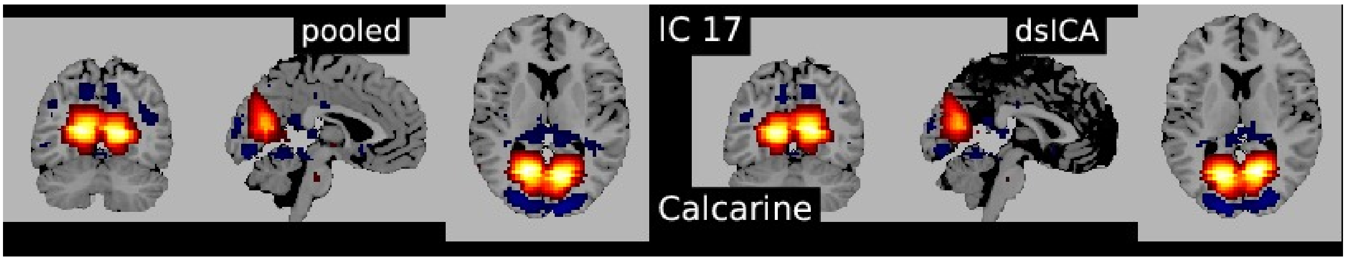

Figure S11. Activation in the Calcarine region of the brain, estimated by pooled ICA and dgICA

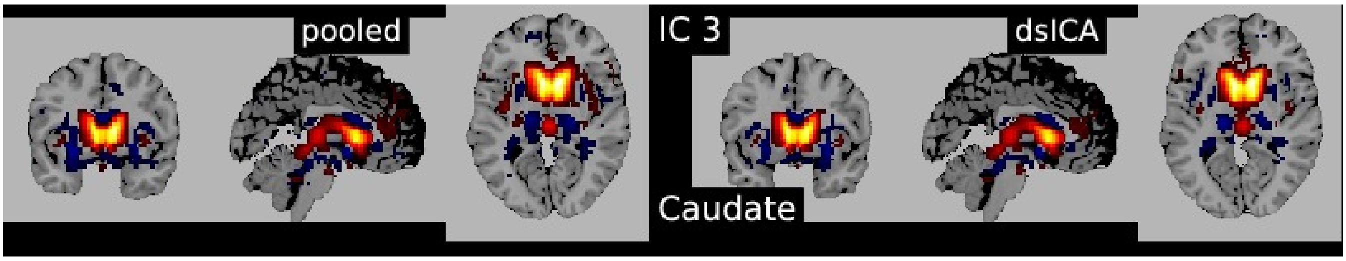

Figure S12. Activation in the Caudate region of the brain, estimated by pooled ICA and dgICA

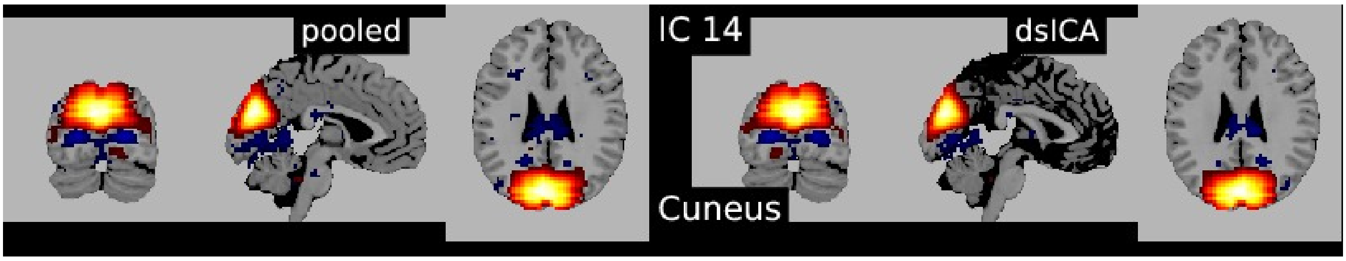

Figure S13. Activation in the Cuneus region of the brain, estimated by pooled ICA and dgICA

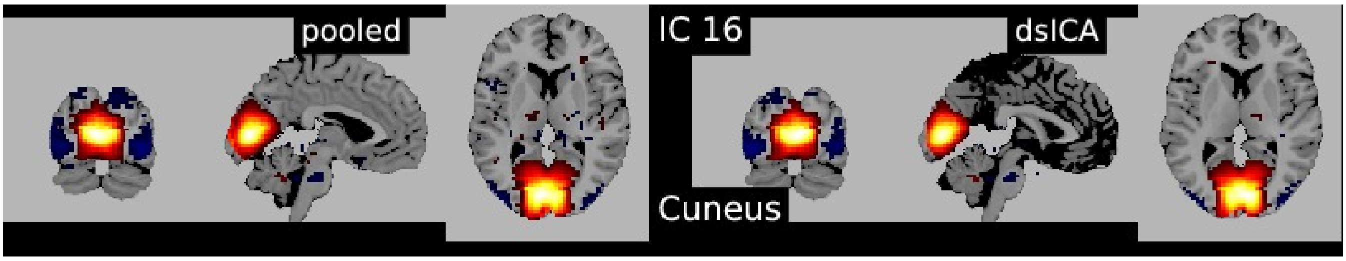

Figure S14. Activation in the Cuneus region of the brain, estimated by pooled ICA and dgICA

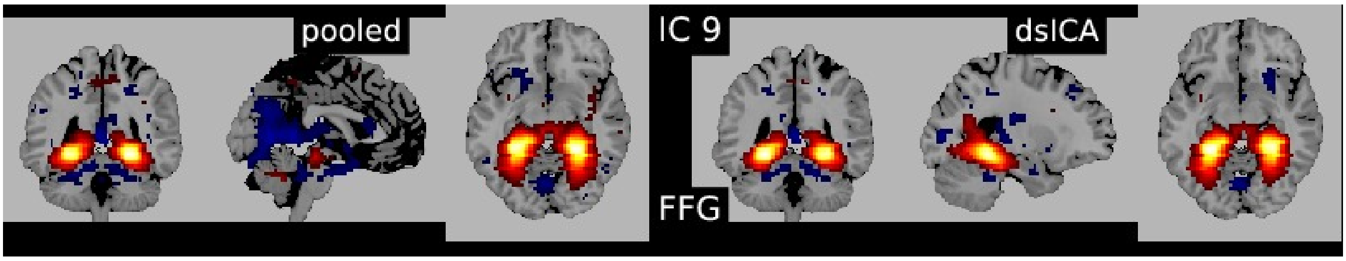

Figure S15. Activation in the FFG region of the brain, estimated by pooled ICA and dgICA

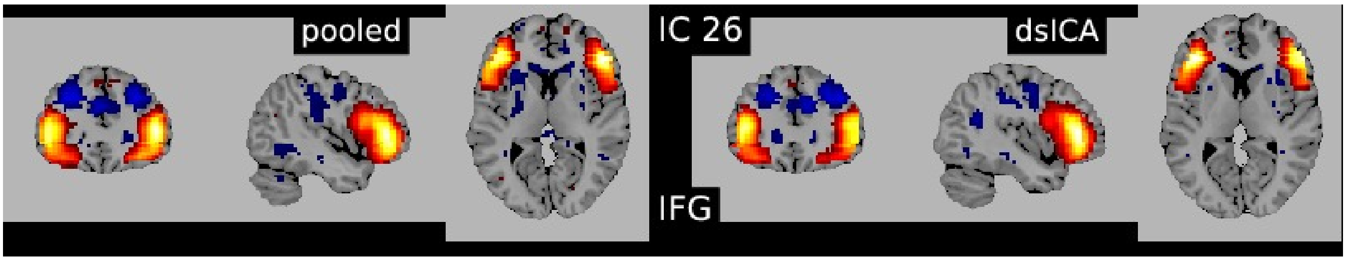

Figure S16. Activation in the IFG region of the brain, estimated by pooled ICA and dgICA

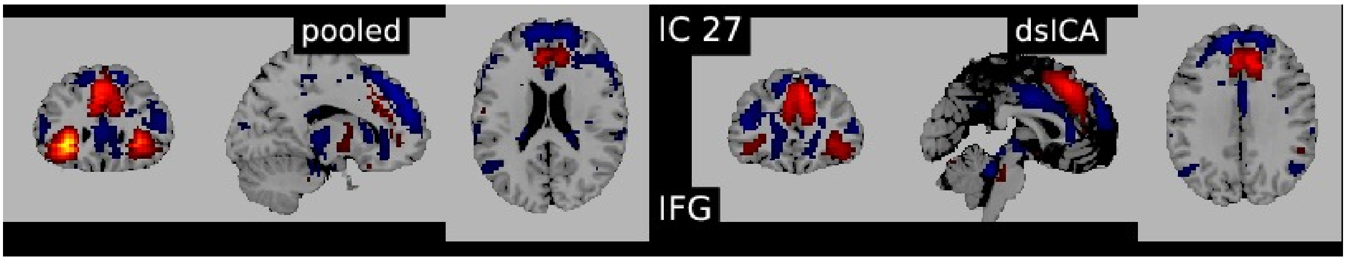

Figure S17. Activation in the IFG region of the brain, estimated by pooled ICA and dgICA

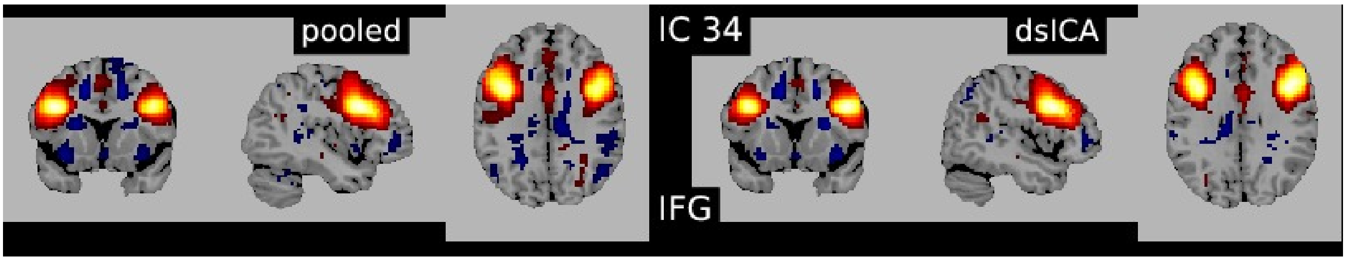

Figure S18. Activation in the IFG region of the brain, estimated by pooled ICA and dgICA

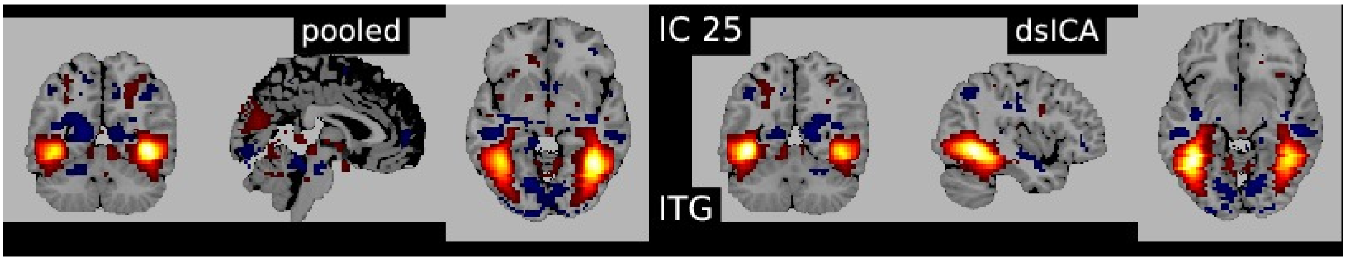

Figure S19. Activation in the ITG region of the brain, estimated by pooled ICA and dgICA

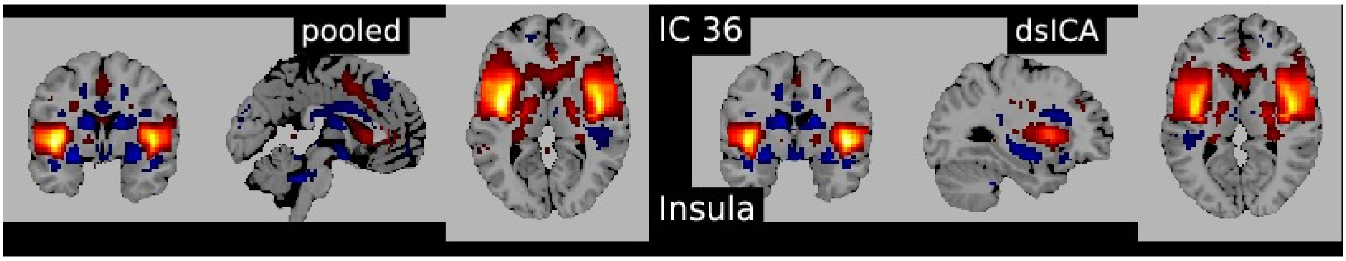

Figure S20. Activation in the Insula region of the brain, estimated by pooled ICA and dgICA

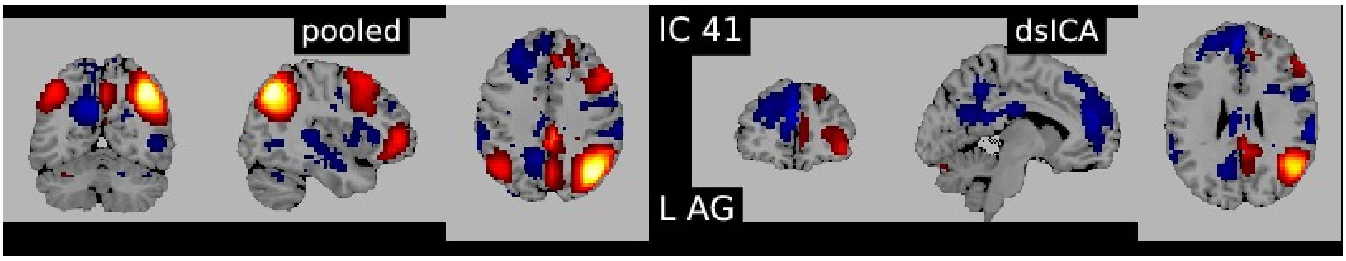

Figure S21. Activation in the L_AG region of the brain, estimated by pooled ICA and dgICA

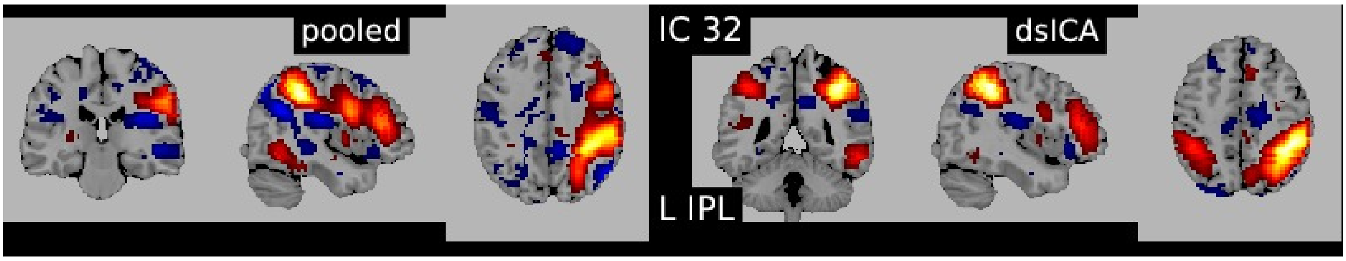

Figure S22. Activation in the L_IPL region of the brain, estimated by pooled ICA and dgICA

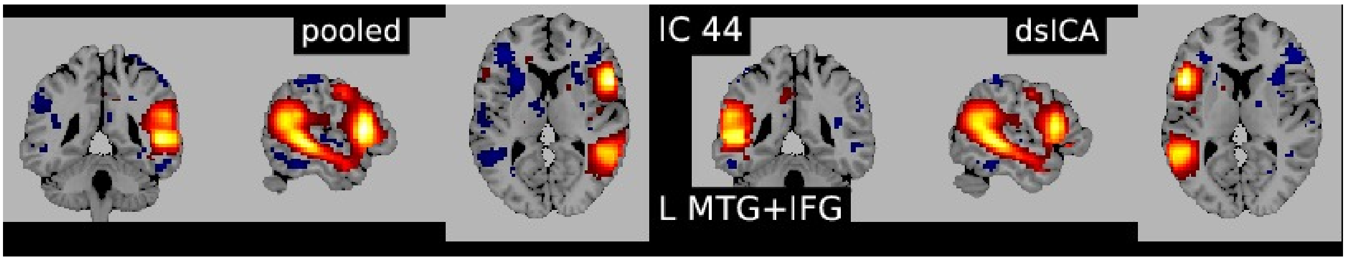

Figure S23. Activation in the L_MTG+IFG region of the brain, estimated by pooled ICA and dgICA

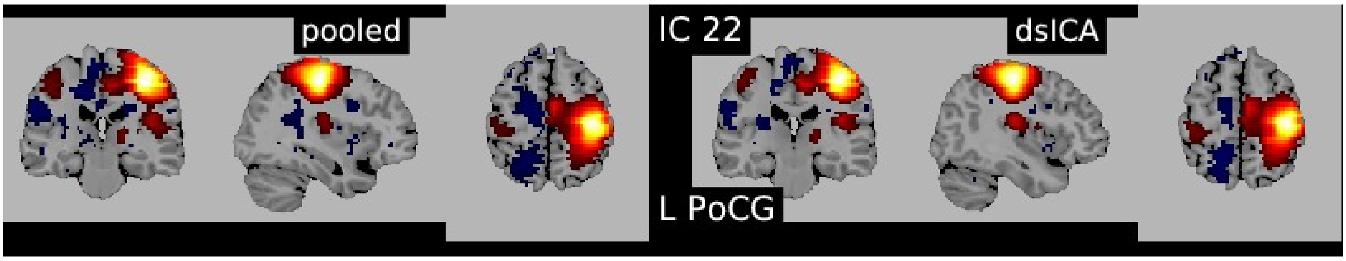

Figure S24. Activation in the L_PoCG region of the brain, estimated by pooled ICA and dgICA

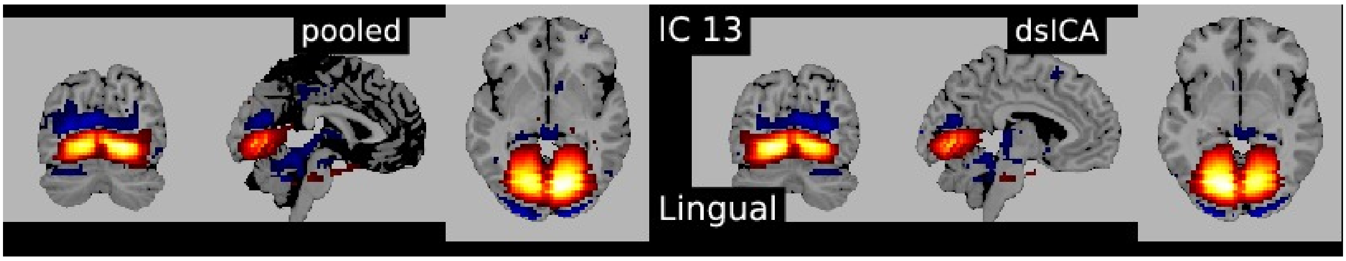

Figure S25. Activation in the Lingual region of the brain, estimated by pooled ICA and dgICA

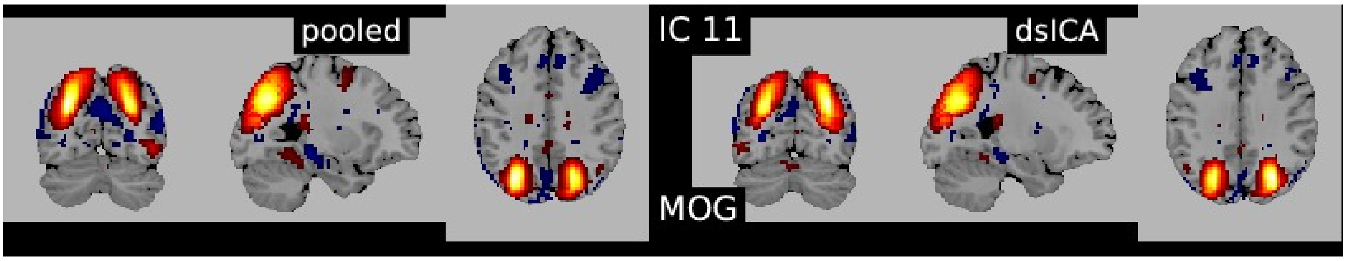

Figure S26. Activation in the MOG region of the brain, estimated by pooled ICA and dgICA

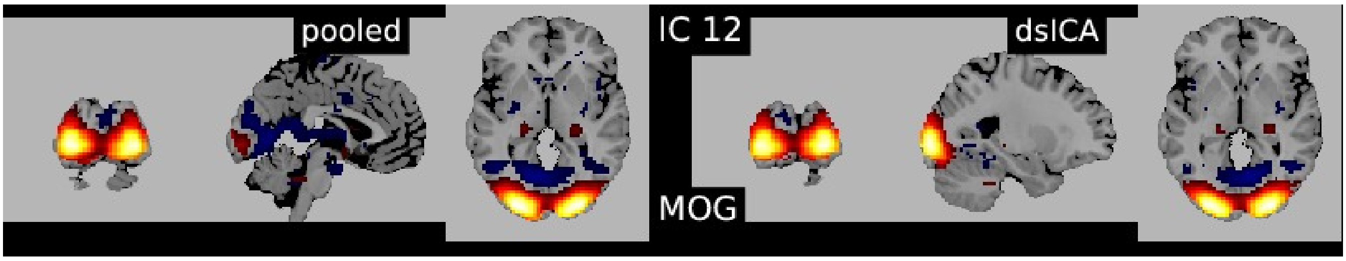

Figure S27. Activation in the MOG region of the brain, estimated by pooled ICA and dgICA

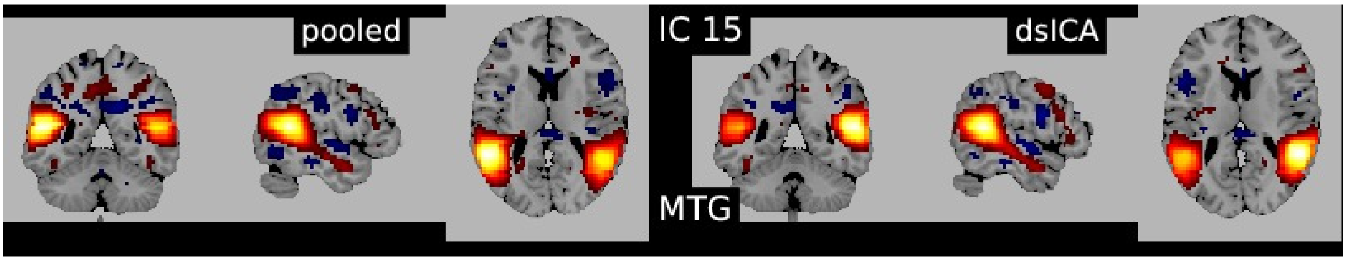

Figure S28. Activation in the MTG region of the brain, estimated by pooled ICA and dgICA

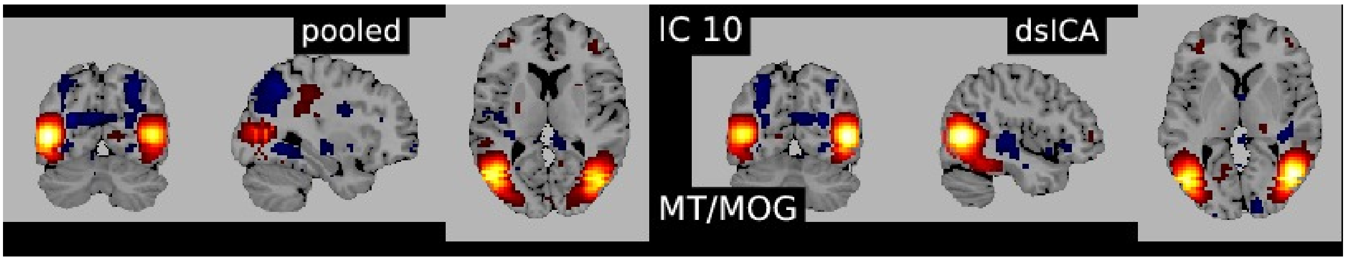

Figure S29. Activation in the MT_MOG region of the brain, estimated by pooled ICA and dgICA

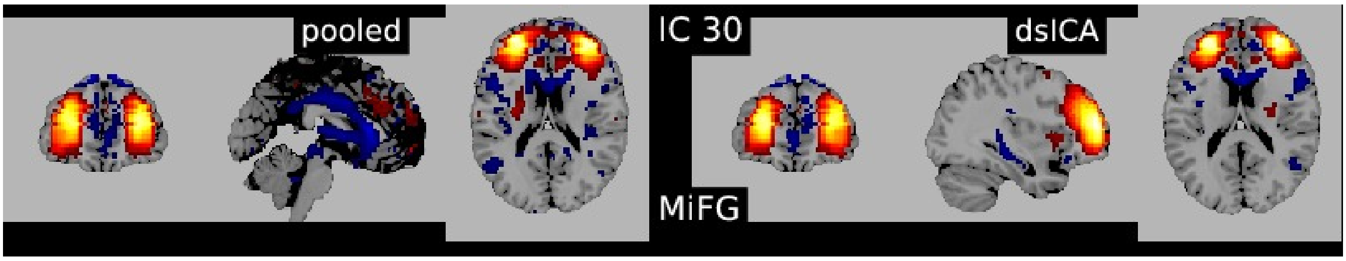

Figure S30. Activation in the MiFG region of the brain, estimated by pooled ICA and dgICA

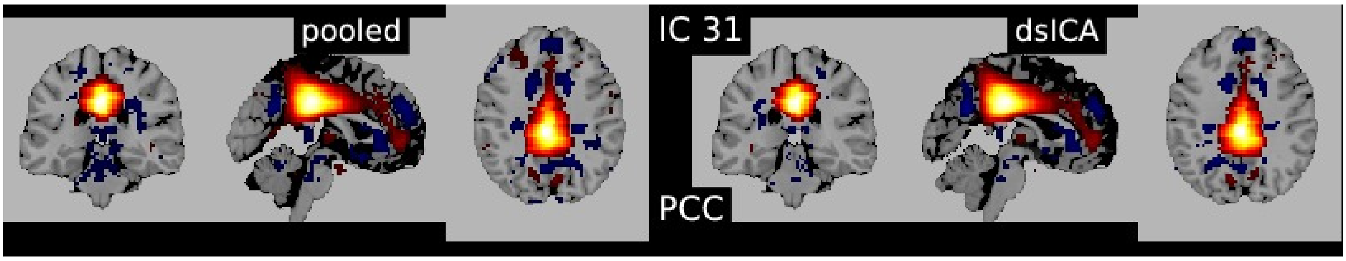

Figure S31. Activation in the PCC region of the brain, estimated by pooled ICA and dgICA

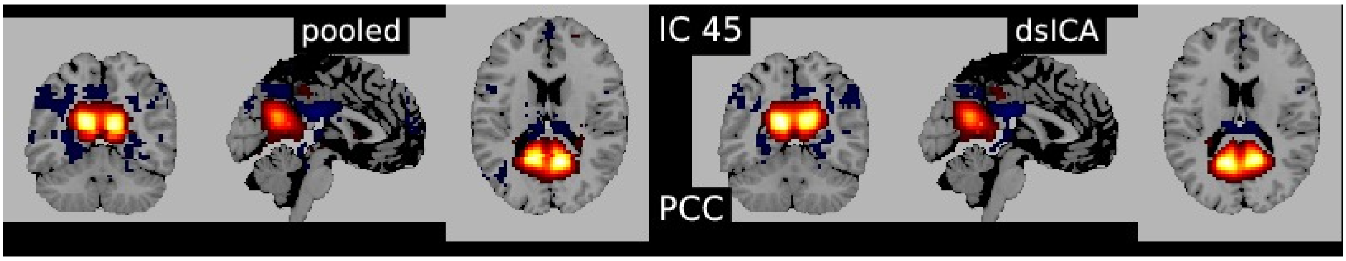

Figure S32. Activation in the PCC region of the brain, estimated by pooled ICA and dgICA

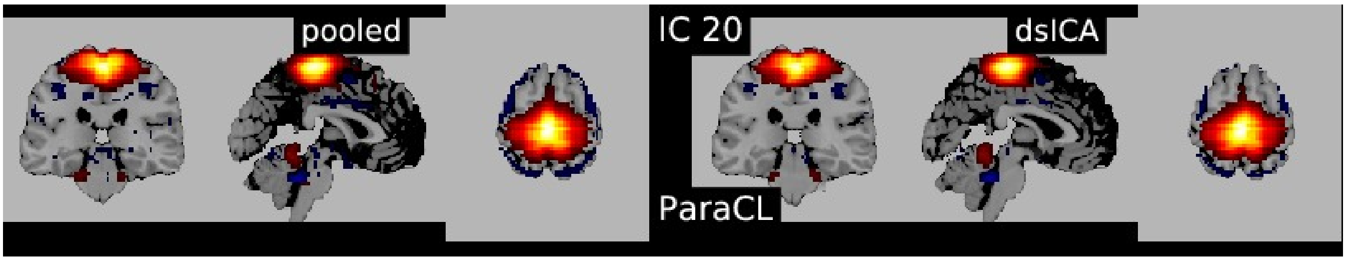

Figure S33. Activation in the ParaCL region of the brain, estimated by pooled ICA and dgICA

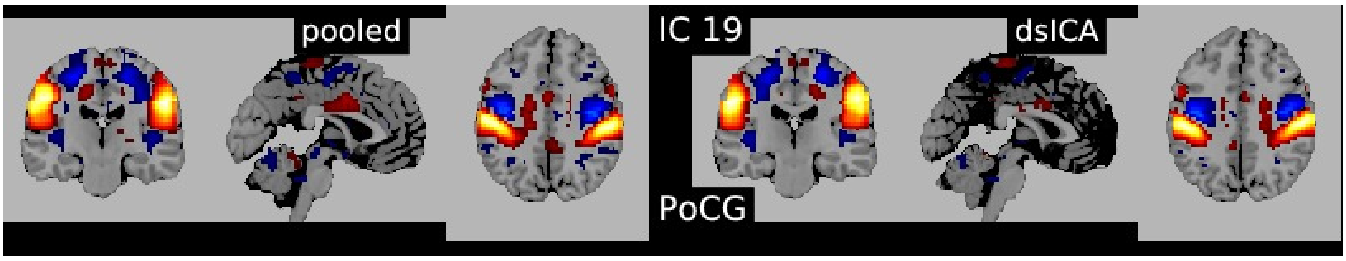

Figure S34. Activation in the PoCG region of the brain, estimated by pooled ICA and dgICA

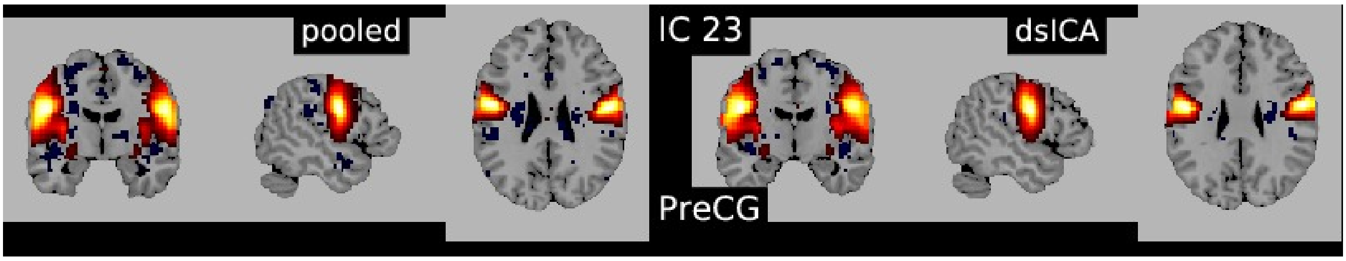

Figure S35. Activation in the PreCG region of the brain, estimated by pooled ICA and dgICA

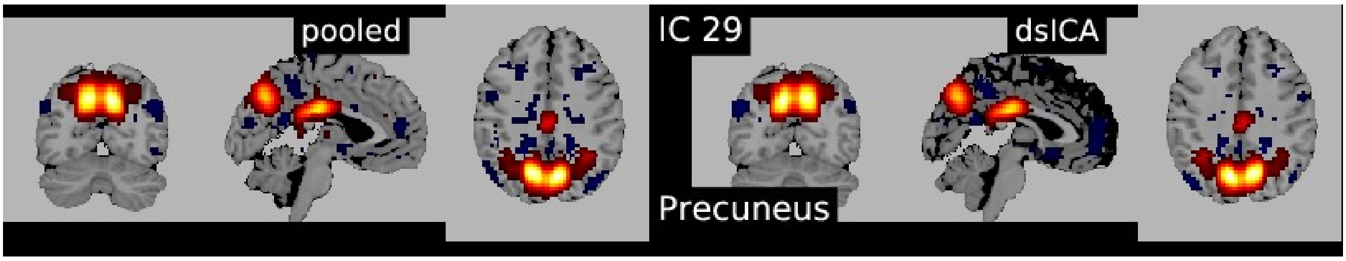

Figure S36. Activation in the Precuneus region of the brain, estimated by pooled ICA and dgICA

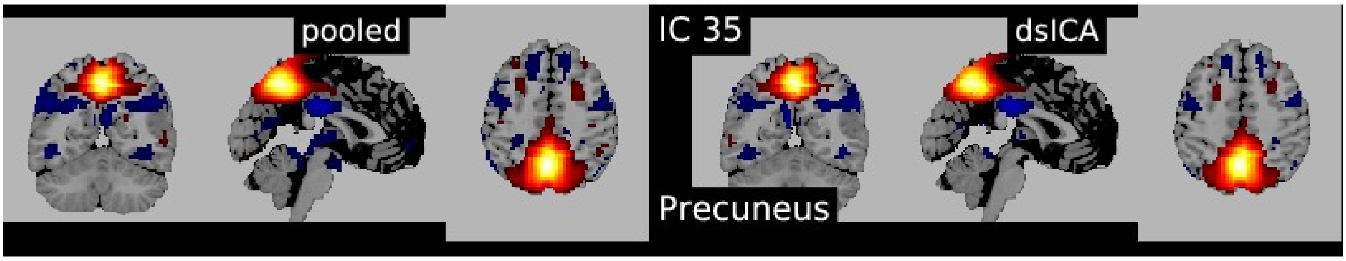

Figure S37. Activation in the Precuneus region of the brain, estimated by pooled ICA and dgICA

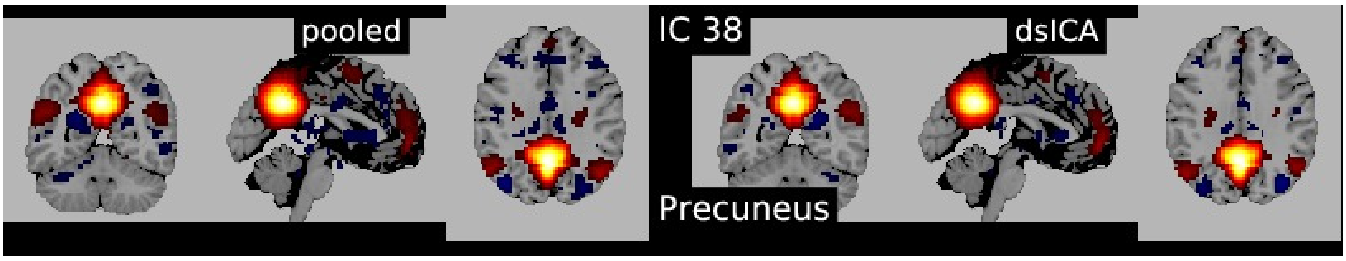

Figure S38. Activation in the Precuneus region of the brain, estimated by pooled ICA and dgICA

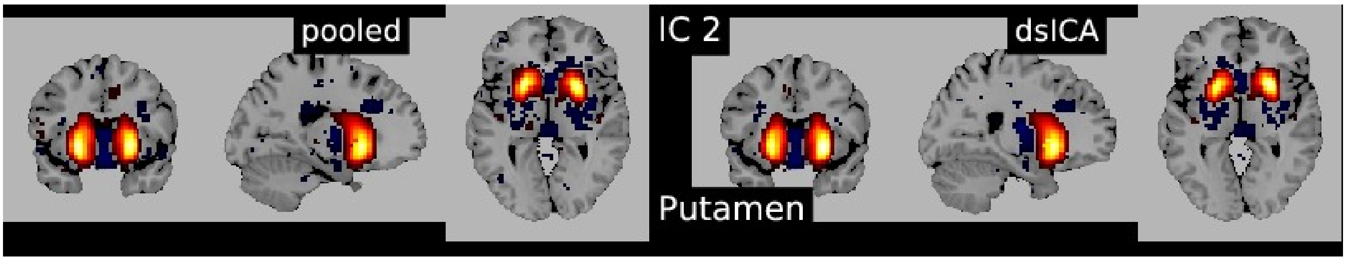

Figure S39. Activation in the Putamen region of the brain, estimated by pooled ICA and dgICA

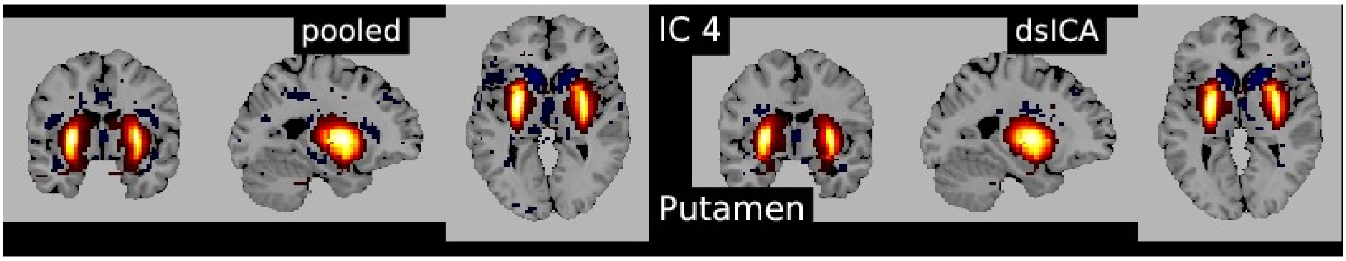

Figure S40. Activation in the Putamen region of the brain, estimated by pooled ICA and dgICA

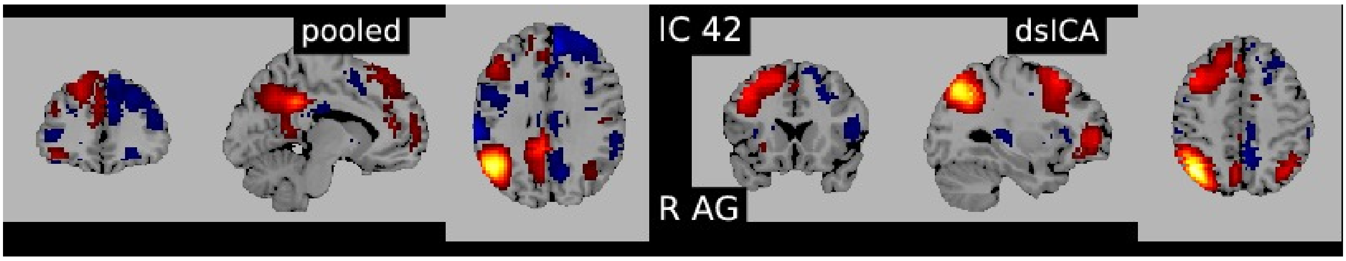

Figure S41. Activation in the R_AG region of the brain, estimated by pooled ICA and dgICA

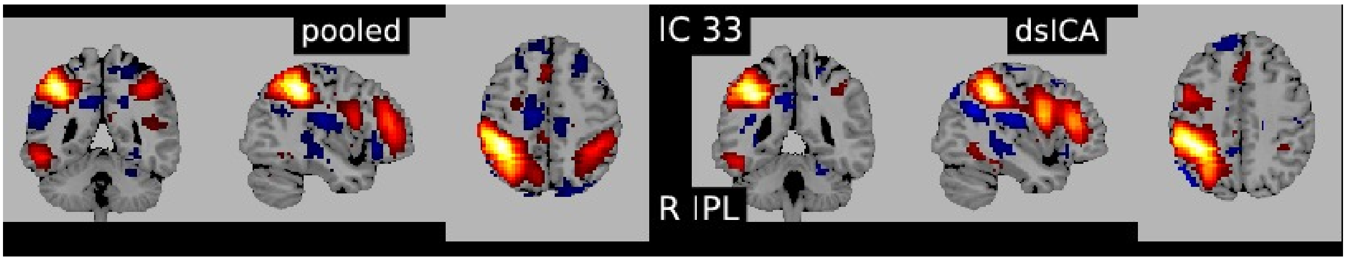

Figure S42. Activation in the R_IPL region of the brain, estimated by pooled ICA and dgICA

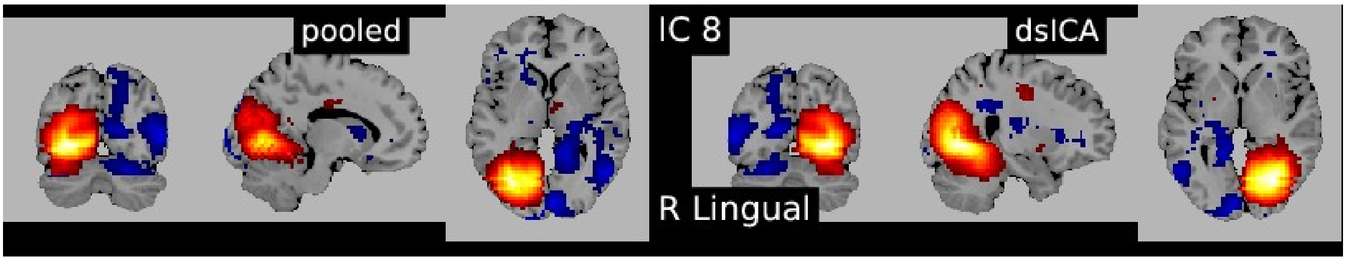

Figure S43. Activation in the R_Lingual region of the brain, estimated by pooled ICA and dgICA

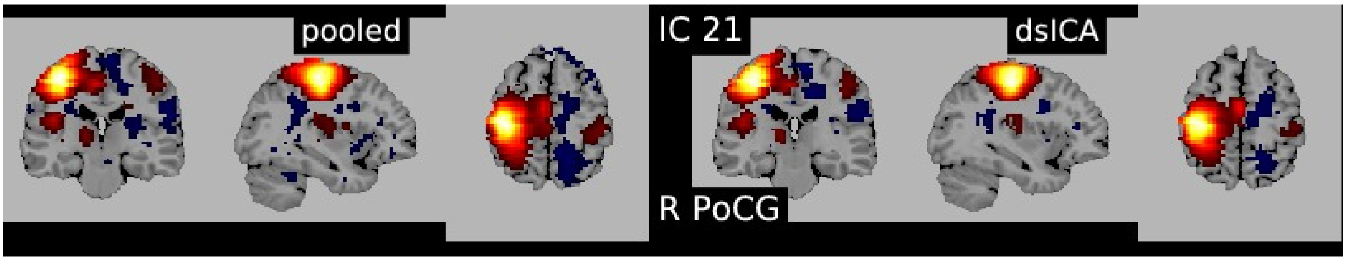

Figure S44. Activation in the R_PoCG region of the brain, estimated by pooled ICA and dgICA

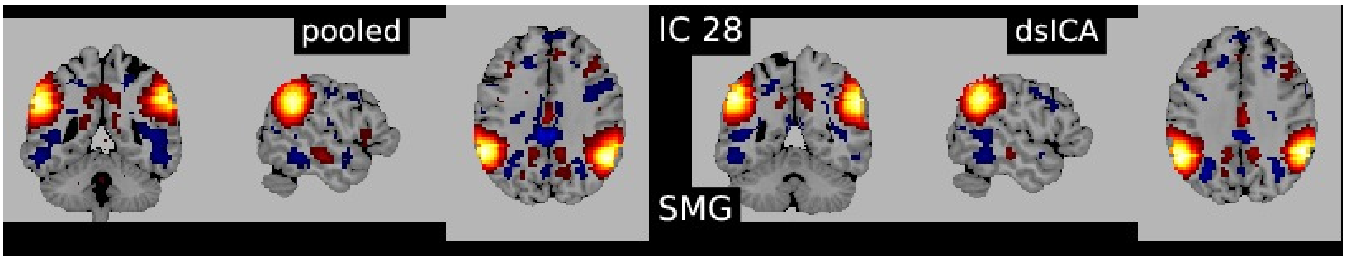

Figure S45. Activation in the SMG region of the brain, estimated by pooled ICA and dgICA

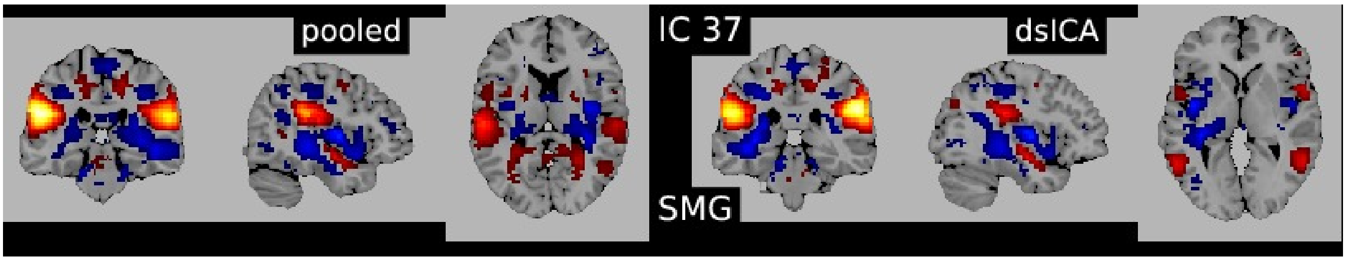

Figure S46. Activation in the SMG region of the brain, estimated by pooled ICA and dgICA

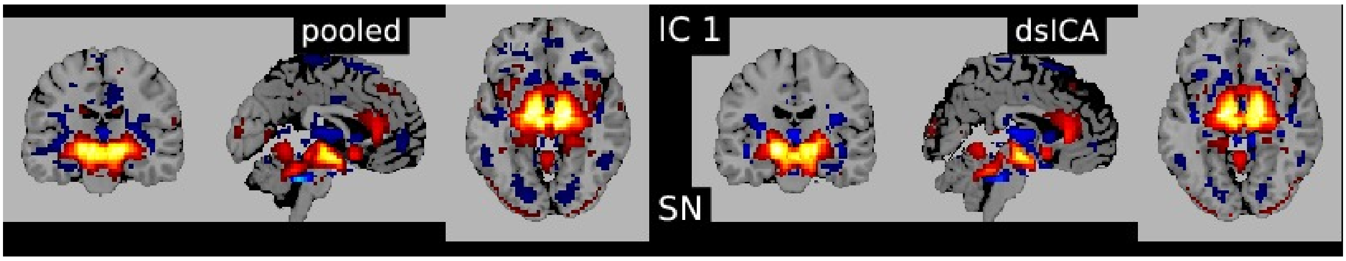

Figure S47. Activation in the SN region of the brain, estimated by pooled ICA and dgICA

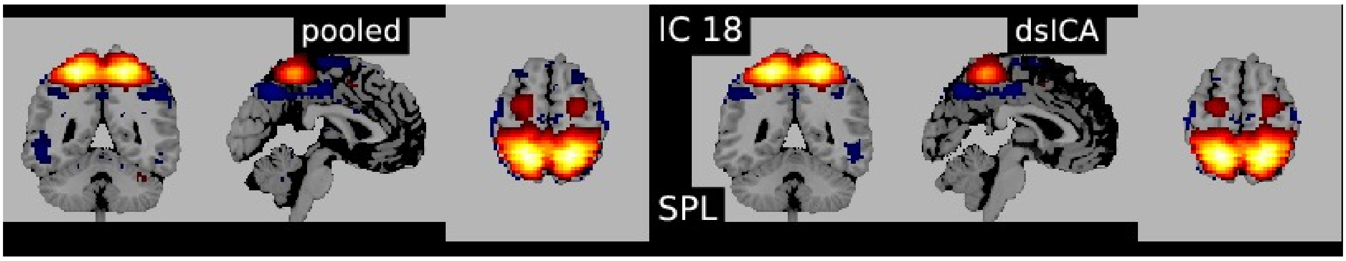

Figure S48. Activation in the SPL region of the brain, estimated by pooled ICA and dgICA

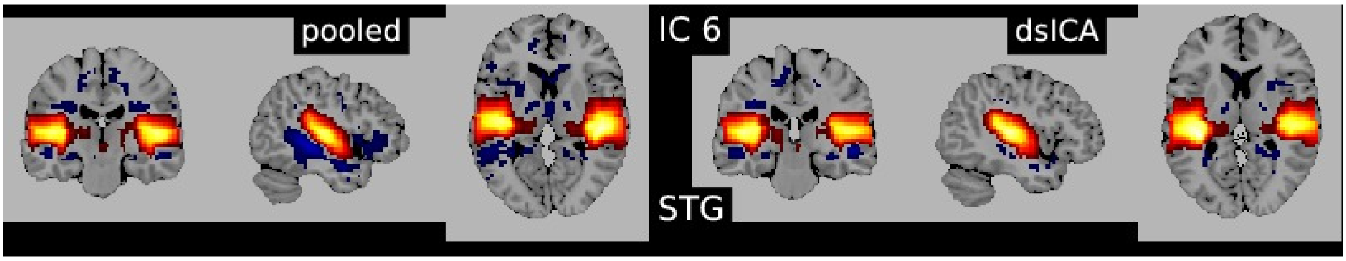

Figure S49. Activation in the STG region of the brain, estimated by pooled ICA and dgICA

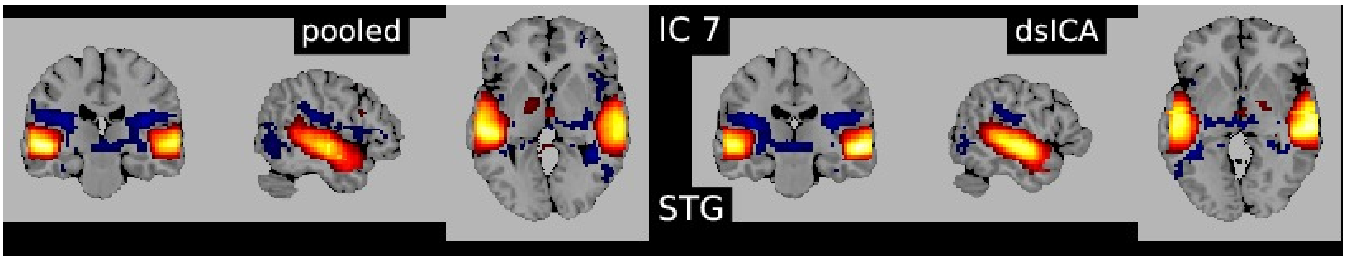

Figure S50. Activation in the STG region of the brain, estimated by pooled ICA and dgICA

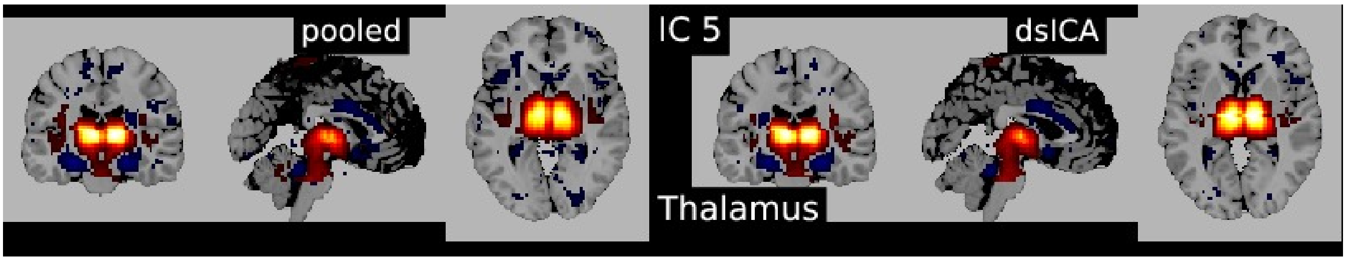

Figure S51. Activation in the Thalamus region of the brain, estimated by pooled ICA and dgICA

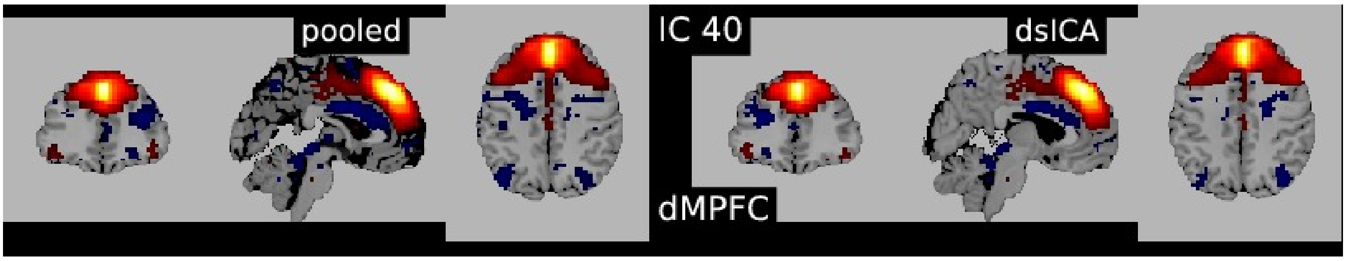

Figure S52. Activation in the dMPFC region of the brain, estimated by pooled ICA and dgICA

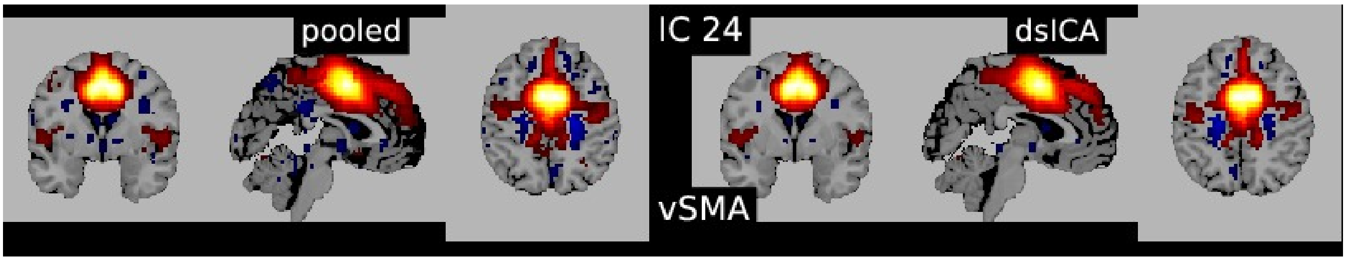

Figure S53. Activation in the vSMA region of the brain, estimated by pooled ICA and dgICA
